# Network structure reveals patterns of legal complexity in human society: The case of the Constitutional legal network

**DOI:** 10.1371/journal.pone.0209844

**Published:** 2019-01-23

**Authors:** Bokwon Lee, Kyu-Min Lee, Jae-Suk Yang

**Affiliations:** 1 Moon Soul Graduate School of Future Strategy, Korea Advanced Institute of Science and Technology, Daejeon, Korea; 2 Ministry of Strategy and Finance, Sejong Government Complex, Sejong, Korea; University of Technology Sydney, AUSTRALIA

## Abstract

Complexity in nature has been broadly found not only in physical and biological systems but also in social and economic systems. Although many studies have examined complex systems and helped us understand real-world complexity, the investigation to the legal complexity has not been thoroughly investigated. Here we introduce a novel approach to studying complex legal systems using complex network approaches. On the basis of the bipartite relations among Constitution articles and Court decisions, we built a complex legal network and found the system shows the heterogeneous structure as generally observed in many complex social systems. By treating legal networks as unique political regimes, we examine whether structural properties of the systems have been influenced as the society changes, or not. On one hand, there is a core structure in all legal networks regardless of any social circumstances. On the other hand, with relative comparison among different regimes’ networks, we could identify characteristic structural properties that reveal their identity. Our analysis would contribute to provide a better understanding of legal complexity and practical guidelines for use in various legal and social applications.

## Introduction

Complexity in physical and biological systems originates from emergent behaviour which is different from the simple sum of individual behaviours [1–3]. Human society has multiple layers of complexity ranging from individual people, groups, and organizations to international relations and economics [4–7]. In many cases, such as complex patterns in the dynamics of pedestrian crowds [8], strategic actions among agents in complex organizations [9], and systemic risks in global financial systems [10], emergent behaviour arises from a myriad of interactions among a large number of agents. Along with various frameworks for modeling complex systems, the complex network has successfully provided theoretical backgrounds and practical methods to investigate real-world complexity with abstracting individual agents as nodes and their interactions as links [11–17]. Especially, complex network theory reveals the topological and dynamic patterns underlying complex systems irrespective of details and characteristics, which implies the universality in nature [18–20].

Modern human societies are based on fundamental principles reflected in laws [21, 22]. Laws regulate human behaviours and, occasionally, provoke behavioural changes. They also provide a fundamental framework for political and economic structures. Furthermore, as environments and societal norms change, laws are altered. For example, when new technology is introduced to a society, new rules are required to regulate activities associated with this technology. Therefore, the existence of feedback loop between laws and society would justify the point of view of considering legal systems as complex systems [23, 24]. Furthermore, the legal complexity arises from the intrinsic attributes of the environments within which laws are enacted. When laws are made, applied or interpreted, many kinds of actors are involved. For example, the national assembly, government, and various interest groups participate in law-making. Judges, lawyers, and jurists interpret those laws. In addition, the legal system has its own complex hierarchy. The Constitution holds a superior position in the legal hierarchy. Acts hold the next position. New Acts take precedence over old Acts. Some compulsory codes or regulations subsume other codes or regulations [25–27]. These external and internal characteristics increase the complexity of legal systems. Thus, a complex system approach would be strongly required to understand the complex nature of legal systems. However, there have been only a few pioneering studies [23–30] that have drawn limited attention by the complexity science field.

In this paper, we introduce a novel approach to the analysis of legal systems via complex network theory. By considering the legal system as a complex network made up of different legal articles, we investigate the longstanding issue of the relationship between laws and society [21]. The autonomy of law has been a fundamental social question since the famous 19th-century debates [31]. Some scholars insisted that the law enjoys autonomy from economic and social surroundings [32]. However, others underlined the dependence of the law on society [21]. To apply the complex network approach to our research question, however, two preceding conditions should be met: (1) The components of legal systems have been unchanged for a long time, but (2) the social circumstances around them have changed considerably. Under these conditions, if the legal network structure has remained the same, even though the surrounding society has changed, the autonomy of the law would be supported. On the other hand, if distinctive structural properties appear in different periods, the law would seem to be dependent on society. Based on this background, the Constitution of the Republic of Korea would be a relevant material by which we can study the autonomy of the law as the following aspects: (1) the Constitution has not changed during the last 30 years after it was enforced in 1988 [33] (see S1 Appendix for more details). (2) Korean society has experienced dramatic political, economic, and cultural changes during the last 30 years. In politics, an authoritarian military regime turned into a free democratic one (see S1 Appendix for more details). Economically, Korea moved from a middle-income country to a developed one. In terms of culture, deeply rooted Confucianism has faded away and a more diversified, westernized culture has become dominant.

**Fig 1:**
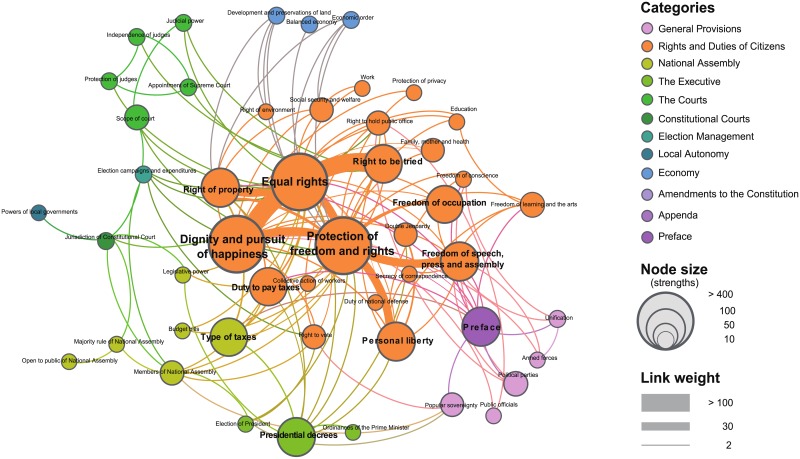
The backbone of the Constitutional legal network (CLN). Each node indicates a Constitutional article, and links are the co-cited relations regarding same decisions. Node sizes are scaled according to strength (Eq 7) and the thickness of each link denotes the number of shared citations between two articles. All articles (nodes) are included in one of 12 categories of the Constitution. Thus, we present the information as colors of different nodes. Since the original CLN is too dense for direct presentation, we use the backbone extraction algorithm [41] for better visualization. One could find the central core articles in the CLN such as “Equal rights”, “Dignity and pursuit of happiness” and “Protection of freedom and rights.”

First, we collected all historical data regarding decisions made by the Constitutional Court of Korea for the 27 years from 15th January 1989 to 24th November 2016 [34]. Among these decisions, we selected 1057 cases that the Court deemed unconstitutional since only those cases contain citations of Constitution articles. From these data, we extracted a bipartite network [35–37] among decisions and articles, and then constructed the Constitutional Legal Network (CLN) by the projection to the articles. The CLN constists of 132 Constitution articles as nodes and co-cited relations among the articles as links (see S1 Appendix for more details). The weight of each link indicates the number of co-cited decisions between two articles, so the CLN could be modeled as a complex weighted network [38–40]. We visualized the CLN using a backbone extraction algorithm for weighted networks [41] (Fig 1). Note that the algorithm was only used for better visualization due to the high link density of the CLN, while all the other network analysis in this study was performed on the whole network.

Through network visualization, we identified some nontrivial topological properties of the CLN (Fig 1), as follows: (i) During the Court’s decision, articles were not selected at random; however, only a few articles, such as “Equal rights”, “Dignity and pursuit of happiness”, and “Protection of freedom and rights” were frequently cited (Fig 1). This implies the existence of hubs, as found in many real-world complex networks [12, 16, 42], which would play a central role in the legal system. (ii) Moreover, the hub nodes seem more likely to be connected to each other rather than to other periphery nodes. This property implies the existence of a core structure originating from the rich-club effect [43, 44]. In our next analysis, we focus on verifying the existence of core structures in the CLN and confirming the influence of changes in social circumstances on the structural properties of the network.

## Materials and methods

### Network randomization

In this study we introduce two types of the null model to verify the statistical significance of the main results from the original network. For the type I null model, we consider the totally random selection of articles in the process of making every decision. This randomization can be conceptualized as a random rewiring of the bipartitie relations [37] between articles and decisions, while only preserving the number of articles in each decision.

The type II null model is constructed by the successive rewiring of two links, preserving the strength of each article. This method preserves the strength sequence of the network (so that the distribution does not change); only the connection patterns change [45]. The specific process is as follows: (i) We randomly choose two different links *l*_*AB*_ and *l*_*CD*_, where *l*_*ij*_ denotes the link connecting nodes *i* and *j*. (ii) The weight of links *w*_*AB*_ and *w*_*CD*_ are decreased by *w*_*m*_, where *w*_*m*_ is the minimum link weight of the original network. If *w*_*AB*_ (or *w*_*CD*_) is the same as *w*_*m*_, then link *l*_*AB*_ (or *l*_*CD*_) would be removed. (iii) We then increase linkweights *w*_*AD*_ and *w*_*BC*_ by *w*_*m*_. If no links exist between nodes *A* and *D*, then a new link is created by this process. (iv) We continue this process until the number of successive rewirings exceeds the given number. This type of randomization can correspond to the link exchanges in unweighted networks [46, 47].

### z-score observation

The z-score of each category (*z*_*c*_), which shows the statistical significance of the observed data, is calculated as

$$\begin{array}{c} z_{c}=\frac{\langle S_{\text{data},c}\rangle -\langle S_{\text{random},c}\rangle }{\sigma_{\text{random},c}}. \end{array}$$ (1)

where 〈*S*_data,c_〉 is the average value of the strength in category *c* observed from real data and 〈*S*_random,c_〉 and *σ*_random,c_ are obtained from randomized samples of the type I null model.

### Rich-club coefficient

The rich-club coefficient of the weighted network is defined by the following equation:

$$\begin{array}{c} \phi_{w}\left(s\right)=\frac{2E_{>s}}{n_{>s}\left(n_{>s}-1\right)} \end{array}$$ (2)

where *E*_>*s*_ is the number of links that have higher strength than the value of *s* and *n*_>*s*_ is the number of nodes, the strength of which is larger than *s* [45]. The normalized rich-club coefficient is obtained by

$$\begin{array}{c} \rho_{w}\left(s\right)=\frac{\phi_{w}\left(s\right)}{\phi_{w}^{random}\left(s\right)} \end{array}$$ (3)

where *ϕ*_*w*_(*s*) denotes the weighted rich-club coefficient of the original network and $\phi_{w}^{random}\left(s\right)$ is its randomized counterpart obtained in the type II null model.

### s-core decomposition

To identify core nodes in the CLN, we use a generalized *k*-core analysis to weighted networks [48]. The *s*_*n*_-core can be obtained through the iteration of removeing nodes as follows: (i) Choose all nodes with strengths *s*_*i*_ ≤ *s*_*n*−1_ and remove them. (ii) Recalculate the strengths of nodes as a result of the removal. (iii) Repeat i)-ii) until there remain no more nodes with strengths *s*_*i*_ ≤ *s*_*n*−1_ in the network. Note that the *s*-core corresponds to the *k*-core analysis when we consider all link weights identical [48].

### Prevalence measure of nodes and links

The prevalence of nodes can be defined by

$$\begin{array}{c} P_{i}^{r}=n_{i}^{r}/N_{c} \end{array}$$ (4)

where $n_{i}^{r}$ is the number of citations of article *i* in regime *r*, and *N*_*c*_ is the total number of citations of articles in the regime [37]. Then the value of,

$$\begin{array}{c} p_{i}^{r}=P_{i}^{r}-{\langle P_{i}^{r^{\prime }}\rangle }_{r\neq r^{\prime }} \end{array}$$ (5)

represents authenticity, which shows the differences between the prevalence in regime *r* and the average prevalence in other regimes. The prevalence of links can be similarly defined by $P_{ij}^{r}=n_{ij}^{r}/N_{c}$ and the relative prevalence measure of the link $p_{ij}^{r}=P_{ij}^{r}-{\langle P_{ij}^{r^{\prime }}\rangle }_{r\neq r^{\prime }}$.

### Network structure comparison

The CLN of each regime can be considered as a series of network layers. Inspired by the recent development of multiplex network analysis [49–51], we measure the network similarity based on the correlation coefficient,

$$\begin{array}{c} \rho_{AB}=\frac{\sum_{l=1}^{L_{max}}\left(w_{A,l}-{\bar{w}}_{A}\right)\left(w_{B,l}-{\bar{w}}_{B}\right)}{\sqrt{\sum_{l=1}^{L_{max}}{\left(w_{A,l}-{\bar{w}}_{A}\right)}^{2}}\sqrt{\sum_{l=1}^{L_{max}}{\left(w_{B,l}-{\bar{w}}_{B}\right)}^{2}}} \end{array}$$ (6)

for all possible pairs of link weights (*L*_*max*_). *w*_*A*(*B*),*l*_ corresponds to the link weight of *l*-th element in regime A(B)’s CLN and ${\bar{w}}_{A(B)}$ denotes the average link weight of regime A(B) respectively.

## Results

### Legal networks are highly heterogeneous

**Fig 2:**
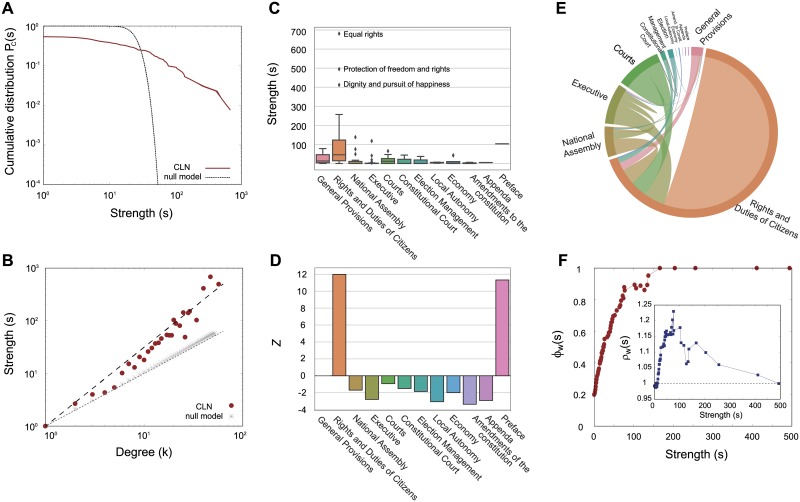
Topological properties of the CLN. (A) The cumulative strength distribution *P*_*c*_(*s*) of the CLN (solid red) compared with the null model counterparts (dotted line). (B) The scaling relation between degrees (*k*) and strengths (*s*) for each node (red dots) compared with the null model (gray stars). The dashed (slope of 1.5) and dotted (slope of 1.0) lines are presented as guides. All data is binned for each degree *k*. (C) A box plot of strengths of articles in each category in the CLN. Significant outliers of high strength (“Equal rights”, “Protection of freedom and rights”, and “Dignity and pursuit of happiness”) are identified as texts. (D) The z-score observation of strengths for each category with 10^4^ samples of null model type I (see Materials and methods). (E) The chord diagram, which presents the net flow between categories of the CLN. (F) The weighted rich-club coefficient *ϕ*_*w*_(*s*) of the CLN and (inset) the normalized one *ρ*_*w*_(*s*) with 10^4^ samples of null model type II (see Materials and methods).

Since we modeled the CLN a complex weighted network, the weighted adjacency matrix *W*_CLN_ can be defined for the mathematical framework. For the main centrality measure, the weighted degree (also referred to as “strength”) is relevant since all links contain information not just about the existence of relations (in standard adjacency matrices) but also of their weights. The strength of a node *i* is defined as

$$\begin{array}{c} s_{i}=\sum_{j}w_{ij} \end{array}$$ (7)

where *w*_*ij*_ denotes a link weight between nodes *i* and *j*. We observe the strengths of all nodes, then obtain the cumulative strength distribution *P*_*c*_(*s*) of the CLN (Fig 2A). Compared with the null model (see Materials and methods for more details), the distribution of the CLN is highly skewed, which implies the existence of a number of highly-cited hub articles along with several periphery nodes of low strengths (Fig 2A). In addition to the distribution, the scaling relation between the nodes’ degrees (*k*) and strengths (*s*)

$$\begin{array}{c} s∼k^{\beta } \end{array}$$ (8)

also reveals the topological properties of a weighted network [39]. Interestingly, the CLN shows nonlinear scaling relations, where the exponent *β* = 1.5 (*β* ∼ 1.569 ± 0.019 with asymptotic standard error of 1.23%) (Fig 2B). This is a property also found in many real-world complex weighted networks like the world-wide air transport network. Clearly, there is a tendency toward concentrating more weighted links to higher-degree articles [39].

Observing the statistical descriptions of different categories, we found that the strengths of the “Rights and Duties of Citizens” (mean strength = 106.6) and the “Preface” [52] (mean strength = 103.0) were much larger (Fig 2C)) than the other 10 categories (mean strength = 11.574). Especially, the three articles, “Equal rights”, “Protection of freedom and rights”, and “Dignity and pursuit of happiness”, are identified as significant outliers with high strength *s* > 400. The dominant position of these two categories is also verified by the z-score calculation (Fig 2D) with samples of the null model (see Materials and methods for more details). Both categories, “Rights and Duties of Citizens” and “Preface” show a large z-score (*z* > 11.0) so that the average value of the strength of those categories are statistically significant compared with random samples from the null model. Moreover, the shared link weights among the “Rights and Duties of Citizens” articles occupy about 55% of all weights (Fig 2E), which implies a high tendency for high-strength articles to be connected to each other. This property can be quantified by the rich-club coefficient [44, 45, 53]. We found a continuously increasing pattern of the coefficient *ρ*^*w*^(*s*) as the strength increases (Fig 2F). Since the increasing pattern was also found in uncorrelated networks, we normalized the measure, producing a randomized version of the CLN by rewiring the edges (null model type II; see Methods for more details). The normalized rich-club coefficient shows a high correlation between medium and rich hubs (Fig 2F, inset), as found in various weighted network structures such as financial and world-trade networks [45, 54, 55]. This result shows a high tendency toward connections among middle- and high-strength articles as well as a high density of link weights among core articles.

### Common core structure exists in different regimes

From the previous analysis, we identified the following nontrivial topological properties of the CLN: (1) the existence of core articles (categories) and (2) a rich-club structure which consists of core articles. Our next step is to investigate the question of whether similar structural characteristics would be found in all different time domains. Since the latest version of the Constitution was revised in the year 1987, there have been six different presidential regimes in South Korea (see S1 Appendix for more details). Therefore, we constructed separate CLN for all 6 regimes ($W_{CLN}^{r},r\in \left\{1,2,\ldots ,6\right\}$) and compared the structural properties of each network.

**Fig 3:**
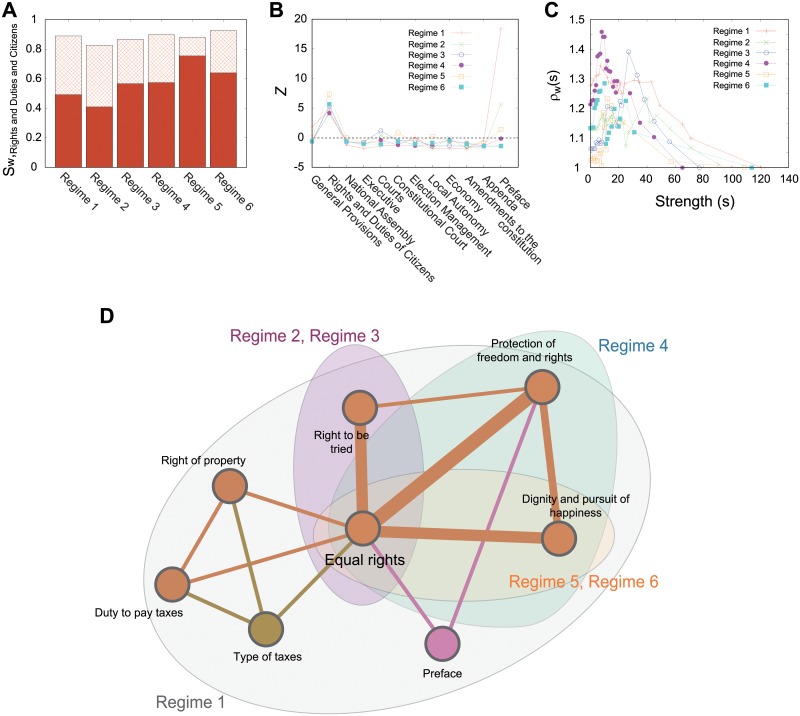
Analysis of the CLN for 6 different regimes and the core structure. (A) Shared link weights of the “Rights and Duties of Citizens” category for 6 regimes. The solid red area denotes the ratio of intra-relations within the category and the checked area is inter-relations with the “Right and Duties of Citizens” and the other categories. (B) Z-score observation, and (C) the normalized rich-club coefficient show a similar statistical pattern for all regimes. (D) The identified core structure of all regimes. Shaded areas denote the results of the *s*-core decomposition for different regimes.

Most importantly, the ratio of linkweights of the category “Rights and Duties of Citizens” to total strengths exceeds 80% for all regimes (Fig 3A). Furthermore, over 40% of strengths concentrate on the intra-relations within the category. Thus, the dominance of the main category has been maintained despite changes in society. The z-score observation of the mean values for strength according to each category for different regimes verifies the dominant position of “Rights and Duties of Citizens” except for the exceptionally high number of citations of the “Preface” in regime 1 (Fig 3B). The special position of the “Preface” in regime 1 can be accepted since the article contains the core values and principles of the Constitution. Therefore, the Courts in the first regime were more likely to cite the article, as the Constitution was in the early stages at that time. The similar patterns among rich-club coefficients (high correlations among medium- and high-strength articles) for all regimes provided another indication of the common structural properties evident in different social circumstances (Fig 3C).

In Fig 3D, we show the fundamental legal structure of each regime (Fig 3D) using the *s*-core decomponsition method (see Materials and methods). For all regimes, the article “Equal rights” takes a central position and is strongly connected to the “Protection of freedom and rights”, “Dignity and pursuit of happiness”, and “Right to be tried” articles that outline the fundamental principles underlying modern democratic society; thus, finding them in the core structure in the CLN of all regimes would support the law’s autonomy to some extent.

### Different regimes show their own identity

Despite the common core structure in the 6 different regimes, their network structures differ. In the s-core decomposition method [48], which was used to identify the core structure for weighted networks, two to three regimes are clustered according to their own core structures (Fig 3D). Specifically, regimes 2 and 3 shared a strong connection between the articles “Right to be tried” and “Equal rights”, whereas regimes 5 and 6 shared the articles “Equal rights” and “Dignity and pursuit of happiness” (Fig 3D). Therefore, the relative comparison among different regimes would reveal the characteristic structure of each regime.

**Fig 4:**
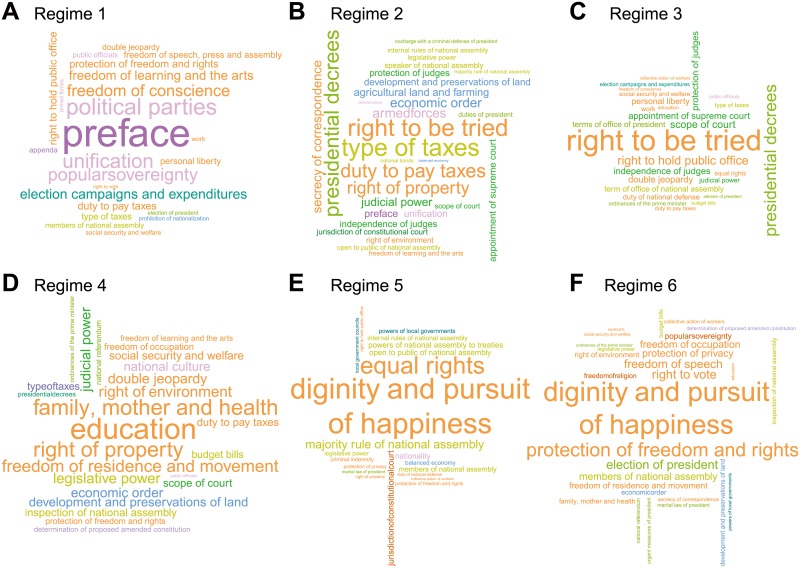
Representative articles for each regime with visualized text groups. Font sizes are scaled according to their nodes’ prevalence measures in the given regime, and the text position is adjusted by the algorithm to produce word clouds. The text is colored according to the categories in which it is included. One would find the keywords representing each regime’s identity.

To perform the relative comparison among different regimes, we measured the relative prevalence, $p_{i}^{r}$ of each article *i* in a regime *r* to quantify how frequently article *i* was cited in a given regime *r* relative to all other regimes (see Materials and methods for more details). A high prevalence of an article in a regime means that the article was more predominant than in any other regimes. We present the result of the measurement of different regimes using a scaled group of texts (known as “word clouds”) for visualization (Fig 4). The larger text represents the more prevalent articles and characteristic identity of the regime. For instance, in regime 1, the articles “Preface”, “Political parties”, and “Unification” have prevalence scores that are from 2 to 10 times larger such that those articles prevail. This can be interpreted as follows: those articles were related to movements of the reorganized government and political parties during that time period (Fig 4A). In regime 4, the high prevalence of “Education” and “Family, mother and health” articles reveals the progressive tendency of the regime (Fig 4D). The common dominant position of the article “Right to be tried” in regimes 2 and 3 and the article “dignity and pursuit of happiness” in regimes 5 and 6 reveals the similar characteristics of these two governments (Fig 4B, 4C, 4E and 4F). More detailed numerical analysis results are presented in the Supplementary Information (S1 Appendix, section 8).

**Fig 5:**
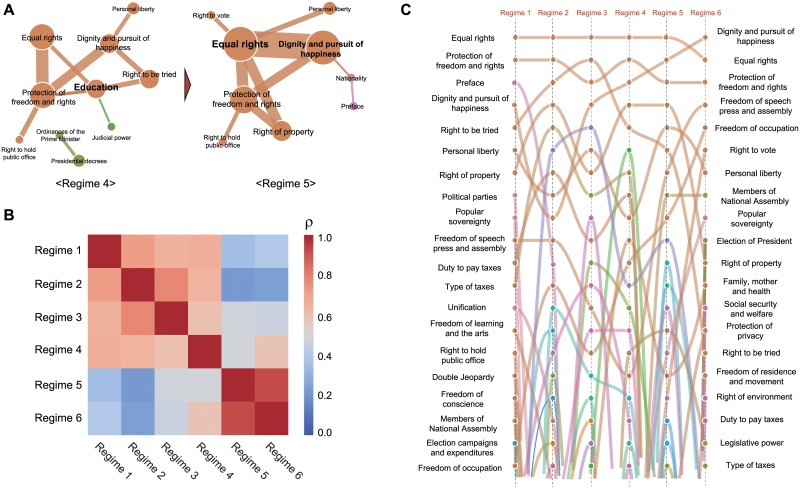
The distinctive properties of each regime. (A) The core structure of each regime’s network identified by link prevalence. Here we present two adjacent regimes (regimes 4 and 5) with markedly different structures within their networks. This indicates that the CLN of each regime would be influenced by the social circumstnaces and identity of the regime. (B) The correlation matrix among the 6 different regimes is depicted by a heat map. We found that some adjacent regimes formed strong clusters, especially regimes 5 and 6, which originiated from the same political parties and share similar government principles. (C) The flow of rank-ordering according to changes in regime from 1 to 6. Each rank is based on each article’s strength in the given regime. We found that the top-rank articles were maintained, but there was a large amount of fluctuation in the middle-ranking articles.

Along with each article’s prevalence, we also measured the relative link prevalence $p_{ij}^{r}$ of each link connecting articles *i* and *j* in a given regime *r* (see Materials and methods). Likewise, in the case of node prevalance, each regime has its own distinctive connections between articles. In Fig 5A, we display the prevalence structure of two adjacent regimes 4 and 5 which shows drastic change. The high prevalence of connections among “Education”, “Equal rights”, “Protection of freedom and rights”, and “Right to be tried” in regime 4 radically changed to a dominant connection among “Equal rights”, “Dignity and pursuit of happiness” and “Protection of freedom and rights” in regime 5. Especially, the prevalence of the link between “Equal rights” and “Dignity and pursuit of happiness” increased from −0.082 in regime 4 to 0.267 in regime 5 which indicates that the strong relation between these two articles in regime 5. The noticeable difference between two adjacent regimes implies that the CLN would strongly reflect the govenment and society’s characteristics. In reality, in the time between regimes 4 and 5, there was a change in political party from progressive to conservative in South Korea (see S1 Appendix for more details). Additionally, we utilized the network structure comparison method based on the correlation coefficient measure for all pairs of link weights among regimes (see Materials and methods). Fig 5B reveals noticeable clustering, especially in regimes 5 and 6 (*ρ* = 0.923). Those two regimes were governed by the same political party and widely considered as sharing many points of philosophy in the government.

Finally, a change in rank-ordering of articles allows us to summarize the main findings of this study (Fig 5C). On one hand, in the case of core articles, such as “Equal rights” (1-1-1-1-1-2 rank change) and “Protection of freedom and rights” (2-2-3-2-3-3 rank change), their dominant position was maintained for all regimes. On the other hand, the abrupt ranking changes such as the case of “Dignity and pursuit of happiness” (from rank 9 in regime 2 to 1 in regime 6) and “Right to be tried” (from rank 2 in regime 3 to rank 15 in regime 6) reflect the change of societal interests. Furthermore, high fluctuation and active changing of rank-ordering of many medium articles reveal the characteristic identity of each regime, quantified as prevalence and network correlation in the previous analysis (Fig 5C).

## Discussion

In this paper, we examined interdependent relationships between law and society, comprehensively applying complex network theory. Building a weighted network consisting of Constitution articles as nodes and their co-cited relations as links, we identified hub articles and a core structure distinguishable from random networks. Hub articles like “Equal rights” and “Protection of freedom and rights” took noticeably cental positions and connected strongly to each other in all different regimes despite differences in social circumstances. The identification of these articles constituting the core structure reveals the time-invariant aspect of the legal system as embodying the fundamental principles of society. In a relative structural comparison among networks of different regimes, however, we also identified the characteristic articles and legal network structure of each regime, providing information about the particular social circumstances in each period. During regime 1, the article “Preface” was found to be predominant. The article states the procedures, core values, and principles of the Constitution and describes the purpose of amendments to it. Right after its enactment in 1988, people needed to understand the core values and principles of the newly amended Constitution; this explains why the “Preface” is more frequently cited in regime 1. The appearance of other prevalent articles such as “Types of taxes” and “Duty to pay taxes” in regime 2 is no doubt related to the financial crisis and budge deficits experienced in South Korea in 1997–1998. In 2016, the Korean President was impeached and suspended. One reason for this impeachment was oppression of freedom of speech. Indeed, from our network analysis of the regime at the time, “Freedom of speech, press and assembly” and “protection of freedom and rights” articles were found to be prevalent.

In modern society, the legal system and its laws form the fundamental structure of society. At the same time, changes in laws reflect the various opinions of citizens. Interdependent relations between laws and society requires complexity science for a full understanding of both the legal system and its relation to society. Our approach of applying complex network theory and conducting an empirical analysis provides a better understanding of the system and the practical applications of legal activities. The structural properties of legal networks would help us quantify political or social changes. For example, in Fig 5B, clustering among different networks represents a quantitative explanation of how one regime is similar to others in the political or social sense. Additionally, a legal network can provide an interpretation or amendment guideline to jurists. Especially when amending certain articles or laws, the overall picture of how close or detached they are from each other would be helpful since the amendment of one article should be accompanied by the consideration of other, highly linked articles. More importantly, our analysis result can provide future guidelines for the amendment of the constitutional system. According to our research, the rank order of some articles (i.e. Dignity and pursuit of happiness or Freedom of occupation) are getting higher while other articles getting lower. This implies that some articles are gaining greater importance in Korean society. When the Constitution is amended, the portion of important articles (i.e. Dignity and pursuit of happiness or Freedom of occupation) should be enlarged and the less important ones be reduced. Moreover, the comprehensive understanding of legal activities and network structures provided in this study may help to elucidate the inherent or systematic problems within society and the support efforts towards designing a better system and improving laws.

Lastly, we discuss the limitations of our analysis and make suggestions for possible future studies. Although we argue that the Constitution of the Republic of Korea is a good example for our research purposes, it is necessary to do a comparative analysis with other countries. Complex network analysis on continental constitutional law such as Germany or Anglo-American constitutional law like the US may have common or different properties with our analysis. These overall comparisons of different systems are expected to provide more profound implications and shed more light on the general principles of legal complexity. On the other hand, research on other forms of legal documents or laws may possibly show the relationship between the legal system and social change. For instance, research on internet related legal documents may capture a better picture of a fast-paced society. Furthermore, the specific mechanism of how legal networks evolve according to social changes remains to be explored. We simply investigated and observed the interrelation between laws and society in a real-world complex system. The general approach of modeling the complex legal system combined with a more profound argument in the sense of jurisprudence would expand our understanding of legal complexity and how it works in our society.

## Supporting information

S1 Appendix Description of the Constitutional legal network. Detailed description of the Constitutional legal network and additional results (PDF). **Fig A**, **The schematic diagram of the Construction of the CLN**. (A) The schematic diagram of the constitutional decision process. From all decisions, we construct the bipartite relations among articles and decisions (B) and generate a projection to the articles to obtain the Constitutional Legal Networks (C) (PDF). **Fig B**, **The full Constitutional legal network**. The full Constitutional legal network. The size of the nodes is adjusted by their degrees and all the links are presented here. Nodes and links are colored according to the categories in which it is included (PDF). **Fig C**, **Cumulative strength distribution**. The cumulative strength distribution (solid line) of the CLN of regime 1 (A) to 6 (F), respectively. The null model counterparts (dotted line) are presented for the comparison (PDF). **Fig D**, **Scaling relation between degrees and strengths**. The scaling relation between degrees and strengths for each node (red dots) compared with the null model (gray stars) of the CLN of regime 1 (A) to 6 (F), respectively. The dashed (slope of 1.5) and dotted (slope of 1.0) lines are presented as guides. All data is binned for each degree *k* (PDF). **Fig E**, **Box plots of strengths of each category**. Box plots of strengths of articles in each category in regime 1 (A) to 6 (F), respectively (PDF). **Fig F**, **Share of link weights between categories**. Share of link weights between categories for the CLN of regime 1 (A) to 6 (F), respectively. Color bars are logarithmically scaled for better visualization (PDF). **Fig G**, **Scatter plots of nodes’ prevalence**. Scatter plots of nodes’ prevalences for each regime. Top 3 articles of high prevalence in each regime are also presented as texts (PDF). **Fig H**, **Core structure of each regime’s CLN**. The core structure of each regime’s network identified by link prevalence. Nodes and links are colored according to the categories in which it is included (PDF). **Fig I**, **Evolution of the giant component**. Evolution of the giant component as the event time progresses. After the increase of the giant component saturated for a while, it grows fast again when regime 4 starts. In regime 4, several articles like “Education” and “Family, mother and health”, which were not paid attention to other regimes, entered the spotlight (PDF). **Fig J**, **Evolution of the sum of strengths of the category**. Evolution of the sum of strengths of each category (PDF). **Fig K**, **Evolution of the sum of strengths of articles 1 to 24**. Evolution of the sum of strengths of articles 1 to 24 (PDF). **Fig L**, **Evolution of the sum of strengths of articles 25 to 48**. Evolution of the sum of strengths of articles 25 to 48 (PDF). **Fig M**, **Evolution of the sum of strengths of articles 49 to 72**. Evolution of the sum of strengths of articles 49 to 72 (PDF). **Fig N**, **Evolution of the sum of strengths of articles 73 to 96**. Evolution of the sum of strengths of articles 73 to 96 (PDF). **Fig O**, **Evolution of the sum of strengths of articles 97 to 120**. Evolution of the sum of strengths of articles 97 to 120 (PDF). **Fig P**, **Evolution of the sum of strengths of articles 121 to 132**. Evolution of the sum of strengths of articles 121 to 132.
(PDF)

S1 Data Node list of the Constitutional legal network. (ZIP)

S2 Data Link list of the Constitutional legal network. (ZIP)

## References

[1] Kauffman SA. The Origin of Order: Self-Organization and Selection in Evolution: Oxford University press, UK; 1993

[2] Gell-Mann M. What is complexity? Complexity. 1995; 1(1):16–19. 10.1002/cplx.6130010105

[3] Vicsek T. Complexity: The bigger picture. Nature. 2002; 418(6894):131 10.1038/418131a 12110869

[4] Schelling TC. Micromotives and Macrobehavior: W.W. Norton, & Company, NY; 1978.

[5] Miller JH, Page SE. Complex Adaptive Systems: An Introduction to Computational Models of Social Life: Princeton University Press, Princeton, NJ; 2007.

[6] Harrison NE (ed.). Complexity in world politics: Concepts and methods of a new paradigm: SUNY Press; 2012.

[7] Battiston S, Farmer JD, Flache A, Garlaschelli D, Haldane AG, Heesterbeek H, Hommes C, Jaeger C, May R, and Scheffer M. Complexity theory and financial regulation. Science. 2016; 351(6275):818–819. 10.1126/science.aad0299 26912882

[8] Helbing D, Johansson A, AI-Abideen HZ. Dynamics of crowd disasters: An empirical study. Physical Review E. 2007; 75(4):046109 10.1103/PhysRevE.75.046109 17500963

[9] Anderson P. Perspective: Complexity Theory and Organization Science. Organization Science. 1999; 10(3):216.

[10] Haldane AG, May RM. Systemic risk in banking ecosystems. Nature. 2011; 469 (7330):351 (2011). 10.1038/nature09659 21248842

[11] Watts DJ, Strogatz SH. Collective dynamics of ‘small-world’ networks. Nature. 1998; 393(6684):440 10.1038/30918 9623998

[12] Barabási AL, Albert R. Emergence of scaling in random networks. Science. 1999; 286(5439):509–512. 10.1126/science.286.5439.509 10521342

[13] Albert R, Jeong H, Barabási AL. Error and attack tolerance of complex networks. Nature. 2000; 406(6794):378 10.1038/35019019 10935628

[14] Albert R, Barabási AL. Statistical mechanics of complex networks. Reviews of Modern Physics. 74(1):47 10.1103/RevModPhys.74.47

[15] Newman MEJ, Barabási AL, Watts DJ. The structure and dynamics of networks: Princeton University Press; 2006.

[16] Caldarelli G. Scale-free Networks: Complex Webs in Nature and Technology: Oxford University Press; 2007.

[17] Cohen R, Havlin S. Complex Networks: Structure, Robustness and Function: Cambridge University Press; 2010.

[18] Goh KI, Kahng B, Kim D. Universal Behavior of Load Distribution in Scale-Free Networks. Physical Review Letters. 2001; 87(27):278701 10.1103/PhysRevLett.87.278701 11800921

[19] Zhou C, Motter AE, Kurths J. Universality in the Synchronization of Weighted Random Networks. Physical Review Letters. 2006; 96(3):034101 10.1103/PhysRevLett.96.034101 16486704

[20] Barzel B, Barabási AL. Universality in network dynamics. Nature Physics. 2013; 9(10):673 10.1038/nphys2741 PMC3852675 24319492

[21] Schiff DN. SOCIO-LEGAL THEORY: SOCIAL STRUCTURE AND LAW. The Modern Law Review. 1976; 39(3):287–310. 10.1111/j.1468-2230.1976.tb01458.x

[22] Unger RM. Law in modern society: The Free Press; 1977.

[23] Ruhl JB, Katz DM. Measuring, Monitoring, and Managing Legal Complexity. Iowa Law Review. 2015; 101:191.

[24] Ruhl JB, Katz DM, Bommarito MJ II. Harnessing legal complexity. Science. 2017; 355(6332):1377–1378. 10.1126/science.aag3013 28360284

[25] Mazzega P, Bourcier D, Boulet R. The network of French legal codes. Proceedings of the 12th international conference on artificial intelligence and law. ACM, 2009.

[26] Bommarito MJ II, Katz DM. A mathematical approach to the study of the united states code. Physica A. 2010; 389(19):4195 10.1016/j.physa.2010.05.057

[27] Katz DM, Bommarito MJ II. Measuring the complexity of the law: the United States Code. Artificial Intelligence and Law. 2014; 22(4):337 10.1007/s10506-014-9160-8

[28] Fowler JH, Johnson TR, Spriggs JF, Jeon S, Wahlbeck PJ. Network analysis and the law: Measuring the legal importance of precedents at the US Supreme Court. Political Analysis. 2007; 15(3):324 10.1093/pan/mpm011

[29] Katz DM, Bommarito MJ II, Blackman J. A general approach for predicting the behavior of the Supreme Court of the United States. PloS ONE. 2017; 12(4): e0174698 10.1371/journal.pone.0174698 28403140 PMC5389610

[30] Koniaris M, Anagnostopoulos I, Vassiliou Y. Network Analysis in the Legal Domain: A complex model for European Union legal sources. Journal of Complex Networks. 2017; 6(2):243 10.1093/comnet/cnx029

[31] Tomlins C. How Autonomous Is Law? Annual Review of Law and Social Science. 2007; 3:45–68. 10.1146/annurev.lawsocsci.3.081806.112741

[32] Weber M. Economy and Society: University of California Press; 1978.

[33] Constitutional Courts of Korea. Available from: http://english.ccourt.go.kr

[34] NATIONAL LAW INFORMATION CENTER. Available from: http://www.law.go.kr/eng/engMain.do

[35] Goh KI, Cusick ME, Valle D, Childs B, Vidal M, Barabási AL. The human disease network. Proceedings of the National Academy of Sciences. 2007; 104(21):8685–8690. 10.1073/pnas.0701361104 PMC1885563 17502601

[36] Saavedra S, Reed-Tsochas F, Uzzi B. A simple model of bipartite cooperation for ecological and organizational networks. Nature. 2009; 457(7228):463 10.1038/nature07532 19052545

[37] Ahn YY, Ahnert SE, Bagrow JP, Barabási AL. Flavor network and the principles of food pairing. Scientific Reports. 2011; 1:196 10.1038/srep00196 22355711 PMC3240947

[38] Yook SH, Jeong H, Barabási AL, Tu Y. Weighted Evolving Networks. Physical Review Letters. 2001; 86(25):5835 10.1103/PhysRevLett.86.5835 11415370

[39] Barrat A, Bartheélemy M, Pastor-Satorras R, Vespignani A. The architecture of complex weighted networks. Proceedings of the National Academy of Sciences. 2004; 101:3747–3752. 10.1073/pnas.0400087101 PMC374315 15007165

[40] Newman MEJ. Analysis of weighted networks. Physical Review E. 2004; 70(5):056131 10.1103/PhysRevE.70.056131 15600716

[41] Serrano MA, Boguñá M. Vespignani A. Extracting the multiscale backbone of complex weighted networks. Proceedings of the National Academy of Sciences. 2009; 106(16):6483–6488. 10.1073/pnas.0808904106 PMC2672499 19357301

[42] Newman MEJ. Power laws, Pareto distributions and Zipf’s law. Contemporary Physics. 2005; 46(5):323–351 10.1080/00107510500052444

[43] Zhou S, Mondragon RJ. The rich-club phenomenon in the Internet topology. IEEE Communications Letters. 2004; 8(3):180 10.1109/LCOMM.2004.823426

[44] Colizza V, Flammini A, Serrano MA. Vespignani A. Detecting rich-club ordering in complex networks. Nature Physics. 2006; 2(2):110 10.1038/nphys209

[45] Zlatic V, Bianconi G, Díaz-Guilera A, Garlaschelli D, Rao F, Caldarelli G, On the rich-club effect in dense and weighted networks. The European Physical Journal B. 2009; 67(3):271–275. 10.1140/epjb/e2009-00007-9

[46] Maslov S, Sneppen K. Specificity and stability in topology of protein networks. Science. 2002; 296(5569):910–913. 10.1126/science.1065103 11988575

[47] Maslov S, Sneppen K, Zaliznyak A. Detection of topological patterns in complex networks: correlation profile of the internet. Physica A. 2004; 333:529–540. 10.1016/j.physa.2003.06.002

[48] Eidsaa M, Almaas E. *s*-core network decomposition: A generalization of *k*-core analysis to weighted networks. Physical Review E. 2013; 88(6):062819 10.1103/PhysRevE.88.062819 24483523

[49] Mucha PJ, Richardson T, Macon K, Porter MA, Onnela JP. Community Structure in Time-Dependent, Multiscale, and Multiplex Networks. Science. 2010; 328(5980):876–878. 10.1126/science.1184819 20466926

[50] Menichetti G, Remondini D, Panzarasa P, Mondragón RJ, Bianconi G. Weighted Multiplex Networks. PLoS ONE. 2014; 9(6):e97857 (2014). 10.1371/journal.pone.0097857 24906003 PMC4048161

[51] Min B, Lee S, Lee KM, Goh KI. Link overlap, viability, and mutual percolation in multiplex networks. Chaos Solitons & Fractals. 2015; 72:49–58. 10.1016/j.chaos.2014.12.016

[52] Note that the category “Preface” has only one article of “Preface”.

[53] Opsahl T, Colizza V, Panzarasa P, Ramasco JJ. Prominence and Control: The Weighted Rich-Club Effect. Physical Review Letters. 2008; 101(16):168702 10.1103/PhysRevLett.101.168702 18999722

[54] Gleditsch KS, Expanded trade and GDP data. Journal of Conflict Resolution. 2002; 46(5):712–724.

[55] Hidalgo CA, Klinger B, Barabási AL, Hausmann R. The product space conditions the development of nations. Science. 2007; 317(5837):482–487. 10.1126/science.1144581 17656717

