# Supplementary material for: Network structure reveals patterns of legal complexity in human society: The case of the Constitutional legal network
Source: PLoS One. 2019 Jan 23;14(1):e0209844. doi: 10.1371/journal.pone.0209844 (PMC6343887; doi:10.1371/journal.pone.0209844)
Supplement: S1 Appendix — Detailed description of the Constitutional legal network and additional results (PDF). Fig A, The schematic diagram of the Construction of the CLN. (A) The schematic diagram of the constitutional decision process. From all decisions, we construct the bipartite relations among articles and decisions (B) and generate a projection to the articles to obtain the Constitutional Legal Networks (C) (PDF). Fig B, The full Constitutional legal network. The full Constitutional legal network. The size of the nodes is adjusted by their degrees and all the links are presented here. Nodes and links are colored according to the categories in which it is included (PDF). Fig C, Cumulative strength distribution. The cumulative strength distribution (solid line) of the CLN of regime 1 (A) to 6 (F), respectively. The null model counterparts (dotted line) are presented for the comparison (PDF). Fig D, Scaling relation between degrees and strengths. The scaling relation between degrees and strengths for each node (red dots) compared with the null model (gray stars) of the CLN of regime 1 (A) to 6 (F), respectively. The dashed (slope of 1.5) and dotted (slope of 1.0) lines are presented as guides. All data is binned for each degree k (PDF). Fig E, Box plots of strengths of each category. Box plots of strengths of articles in each category in regime 1 (A) to 6 (F), respectively (PDF). Fig F, Share of link weights between categories. Share of link weights between categories for the CLN of regime 1 (A) to 6 (F), respectively. Color bars are logarithmically scaled for better visualization (PDF). Fig G, Scatter plots of nodes’ prevalence. Scatter plots of nodes’ prevalences for each regime. Top 3 articles of high prevalence in each regime are also presented as texts (PDF). Fig H, Core structure of each regime’s CLN. The core structure of each regime’s network identified by link prevalence. Nodes and links are colored according to the categories in which it is included (PDF). Fig I, Evolution of [file pone.0209844.s001.pdf]

S1 Appendix Supplementary information

Network structure reveals patterns of legal complexity in human society:
The case of the Constitutional legal network
by Bokwon Lee, Kyu-Min Lee, and Jae-Suk Yang

December 20, 2018

Contents

S1 Korean Legislative System **3**
S1.1 Constitution of Korea 3
S1.2 Constitutional judgement act 5

S2 Korean historical regimes **5**

S3 Construction of the CLN **6**

S4 Strength distributions of each regime's CLN **8**

S5 Scaling relations between degrees and strengths in each regime **8**

S6 Strengths of articles in each category for the different regime's CLN **8**

S7 Share of link weights between categories of each regime's CLN **8**

S8 Prevalence measures of each regime **9**
S8.1 Nodes' prevalance 9
S8.2 Links' prevalence 9

S9 Time-dependent analysis of the evolution of the network **9**
S9.1 Giant component evolution 9
S9.2 The evolution of the network centrality of categories 10
S9.3 The evolution of the network centrality of articles 10

List of Figures

A	The schematic diagram of the Construction of the CLN	12
B	The full Constitutional legal network.	13
C	Cumulative strength distribution	14
D	Scaling relation between degrees and strengths	15
E	Box plots of strengths of each category	16
F	Share of link weights between categories	17
G	Scatter plots of nodes' prevalence	18
H	Core structure of each regime's CLN	19
I	Evolution of the giant component	20
J	Evolution of the sum of strengths of the category	21
K	Evolution of the sum of strengths of articles 1 to 24	22
L	Evolution of the sum of strengths of articles 25 to 48	23
M	Evolution of the sum of strengths of articles 49 to 72	24
N	Evolution of the sum of strengths of articles 73 to 96	25
O	Evolution of the sum of strengths of articles 97 to 120	26
P	Evolution of the sum of strengths of articles 121 to 132	27

List of Tables

S1	Periods and political characteristics of each regime	6
S2	Basic network properties of each regime	7

S1 Korean Legislative System

The Korean legislative system consists of the Constitution, Acts and administrative legislations such as Presidential Decree [1]. There is hierarchy in the legislative system. The Constitution is the paramount law. Acts are subordinate to the Constitution. Acts should not be in conflicts with the Constitution. Administrative legislations are subordinate to Acts. The Constitution is amended by national referendum. Acts are determined by voting among members of the National Assembly. Administrative legislations are set by the government [1].

S1.1 Constitution of Korea

Since the Korean government established in 1948, the Constitution has been revised 9 times, and the latest version was amended by Oct. 29, 1987 [2]. The Constitution stipulates fundamental issues such as rights and obligations of people, structure of government, economic system, election management and so on. The Constitution consists of following 12 categories [2].

(1) Preface

(2) Chapter 1 (General Provisions) has 9 articles of Popular sovereignty, Nationality, Territory, Unification, Armed forces, Treaty and aliens, Public officials, Political parties, National culture.

(3) Chapter 2 (Rights and Duties of Citizens) has 30 articles which state Diginity and pursuit of happiness, Equal rights, Personal liberty, Double Jeopardy, Freedom of residence and movement, Freedom of occupation, Freedom from intrusion into residence, Protection of privacy, Secrecy of correspondence, Freedom of conscience, Freedom of religion, Freedom of speech / press and assembly, Freedom of learning and the arts, Right of property, Right to vote, Right to hold public office, Right to petition, Right to be tried, Criminal indemnity, Compensation for damage, Aid from the State, Education, Work, Collective action of workers, Social security and welfare, Right of environment, Family / mother and health, Protection of freedom and rights, Duty to pay taxes, Duty of national defense.

(4) Chapter 3 (National Assembly) has 26 articles of Legislative power, Members of National Assembly, Term of office of National Assembly, Prohibition of concurrent offices of Natonal Assembly, No arrest of members of National Assembly, Protection of votes of National Assembly,

Duties of members of National Assembly, Session of National Assembly, Speaker of National Assembly, Majority rule of National Assembly, Open to public of National Assembly, Not deliberating the same bill, Introduction of bills, Process of bills, Budget bills, Continuing disbursement, Revised budget bill, Increase of expenditure in the budget, National bonds, Type of taxes, Powers of National Assembly to treaties, Inspection of National Assembly, Attending in National Assembly, Removal of State Council members, Internal rules of National Assembly, Impeachment of National Assembly.

(5) Chapter 4 (The Executive) has 35 articles which state Duties of President, Election of President, Next president election, Inauguration oath of President, Terms of office of President, Vacancy of President, National referendum, Treaty powers of President, Commander-in-Chief of the Armed Forces, Presidential decrees, Urgent measures of President, Marital law of President, Appointment of public officials of President, Amnesty, Decoration, Attending of President in National Assembly, Acts of President in writing, Prohibition of concurrent offices of President, No charge with a criminal defense of President, Courteous treament of former Presidents, Prime Minister, Members of State Council, Operation of State Council, Matters of State Council, Advisory Council of Elder Statesmen, National Security Council, Advisory Council on Democratic and Peaceful Unification, National Economic Advisory Council, Appointment of heads of Executive ministries, Ordinances of the Prime Minister, Executive Ministries, Establishment of Board of Audit and Inspection, Composition of Board of Audit and Inspection, Results of Board of Audit and Inspection, Acts related to Board of Audit and Inspection.

(6) Chapter 5 (The Courts) has 10 articles of Judicial power, Composition of Supreme Court, Independence of judges, Appointment of Supreme Court, Term of office of Supreme Court, Protection of judges, Scope of court, Internal rules of Supreme Court, Open to public of courts, Courts-martial.

(7) Chapter 6 (Constitutional Court) has 3 articles of Jurisdiction of Constitutional Court, Term of office of the Justices of Constitutional Court, Decision of Constitutional Court.

(8) Chapter 7 (Election management) has 3 articles which state Composition of election commissions, Powers of election commissions, Election

campaigns and expenditures.

(9) Chapter 8 (Local Autonomy) has 2 articles of Powers of local governments, Local government councils.

(10) Chapter 9 (Economy) has 9 articles which state Economic order, Land and natural resources, Agricultural land and farming, Development and preservations of land, Balanced economy, Consumer protection, Foreign trade, Prohibition of nationalization, Development of science and technology.

(11) Chapter 10 (Amendments to the Constitution) has 3 articles Amendment of Constitution, Annoucement of proposed amended Constitution, Determination of proposed amended Constitution.

(12) Appenda

S1.2 Constitutional judgement act

The Constitutional Court has authority of making decision on whether any legal activity or Act(including administrative legislation) is in violation of the Constitution or not [2]. If certain legal activities are unconstitutional, Constitutional court makes legal decisions by quoting certain articles of the Constitution. The Court procedures are initiated by the request for an adjudication from people, other courts or National Assembly. The Full Bench are convened to review the constitutional cases. The Full Bench consists of all nine Justices. The Full Bench makes final decision on a case by the majority vote. The Constitutional Court does not adjudicate the same cases with previous adjudication [2].

S2 Korean historical regimes

In 1987, the Korean Constitution was amended thanks to June Democracy Movement, which demanded direct election of the President. In 1988, Taewoo Roh became president by the first direct presidential election under amended Constitution. He came from a military general and former member of military coup détat. His regime was considered as military-conservative. He held the presidency from 1988 to 1993. Next, Young-sam Kim was elected as president. He held the presidency from 1993 to 1998. His regime was considered as civil-conservative. During his term, South Korea suffered from Asian financial crisis. Dae-jung Kim was inaugurated as president in

1998. Both Young-sam Kim and Dae-jung Kim had fought for democracy for a long time as political comrades. His regime (1998–2003) was considered as progressive. He is the first president who was elected from opposition party(progressive party) in Korean political history. Next president, Moo-hyun Roh, came from the same party with the former president. His regime, also, was considered as progressive. During his regime (2003–2008), he tried to solve many social issues such as environment or womans rights. From 2008, South Koreas political power went back to conservative party. Myung-bak Lees regime (2008–2013) was considered as conservative. Next president, Geun-hye Park, came from the same party with the former president. Her regime (2013–2017) was considered as conservative. Her presidency was suspended by impeachment. In Table S1, we summarize the periods and political characteristics of each regime.

Time domain	year	Political character
Regime 1	1989 – 1993	Authoritarian (Military)
Regime 2	1994 – 1998	Conservative (Democratic)
Regime 3	1999 – 2003	Progressive (Democratic)
Regime 4	2004 – 2008	Progressive (Democratic)
Regime 5	2009 – 2013	Conservative (Democratic)
Regime 6	2014 – 2016	Conservative (Democratic)

Table S1: The periods and political characteristics of each regime in South Korea since 1989.

S3 Construction of the CLN

We start our research with collecting all historical Constitutional decisions data for 27 years. In civil society, each individual or organization request for adjudication of certain legal activities (Figure A(A)). When a certain legal activitie were identified as unconstitutional, the Constitutional court make a decision by citing some Constitution articles which provide a basis for the decision. Since only the unconstitutional cases quote articles, we choose 1057 decisions and produce the bipartitle network through mapping the relations among decisions and articles (Figure A(B)).

The bipartite network consists of two sets of nodes; articles and decisions. We can generate two directions of projections for each set as well known examples of previous researches like collaboration relations among actors [3]

and scientists [4], human disease [5], organization [6]. The projection of the bipartite relations onto the articles shapes the Constitutional legal network (CLN) of 132 nodes which has undirected and weighted network structure (Figure A(C)). The weight of each link corresponds the number of co-cited decisions by same decisions. The full CLN network has 132 nodes and 496 edges. Since only 71 nodes have non-zero degrees in the CLN, the effective mean degree of the network becomes 13.9 so the network becomes so dense (Figure B). Therefore, for the better visualization, we perform the backbone extraction algorithm [7] in main manuscript. With the simple visualization, we could identify the modular structure and core articles which stands central position of the CLN. The backbone extraction algorithm, however, only used for better visualization and all the network analysis of our research was performed on the full network (Figure B).

Time domain	N	$\langle k \rangle$	$\langle s \rangle$	$\langle C \rangle$	$\phi_{\text{Rights and Duties of Citizens}}$
Regime 1	37	10.65	23.77	0.790	0.691
Regime 2	46	10.04	29.42	0.790	0.618
Regime 3	40	8.45	23.80	0.785	0.717
Regime 4	39	8.62	18.32	0.783	0.736
Regime 5	40	4.75	16.58	0.550	0.817
Regime 6	44	7.82	23.29	0.803	0.783

Table S2: Basic network properties of each regime.

In Section 2, we describe characteristics of Korean historical regimes since 1989. As stated in main manuscript, the Constitution articles of the Republic of Korea has not changed during the last 30 years but the society has experienced dramatic changes. Therefore, we construct CLN for each political regime and compare their structural properties.

In Table S2, we summarize the basic network properties of each regime's CLN. For all CLNs, only about 30% of nodes have non-zero degrees and networks are so dense in the sense of high mean degrees $\langle k \rangle$ and strenghts $\langle s \rangle$. Furthermore, all the CLNs are highly clustered ($\langle C \rangle$) and the considerable rate of linkweights ($\approx 70\%$) are concentrated into the articles of "Rights and Duties of Citizens" category. These observations imply that the strengths of CLN networks are highly concentrated to smalll number of articles rather than equally distributed to all ones.

S4 Strength distributions of each regime's CLN

We observe the cumulative strength distribution $P_c(s)$ of each regime's CLN and compare the distribution with the null model counterparts (Figure C). As we found in main manuscript, the strength distributions of the CLN of each regime are all highly skewed. This implies that there exist a number of highly-cited hub articles in all regimes.

S5 Scaling relations between degrees and strengths in each regime

For all regimes, the scaling relations between degrees and strengths are non-linear ($\beta > 1.0$) (Figure D). This shows that the macroscopic structural properties of CLN are robust regardless of the social (political) environments changes.

S6 Strengths of articles in each category for the different regime's CLN

In main manuscript, we observe the statistical descriptions of different categories in total CLN. Here we perform the same analysis for each different regime (Figure E). As found in the main article, the strengths of the "Rights and Duties of Citizens" articles were much larger than the other categories. Furthermore, the distribution of the categories except for the "Rights and Duties of Citizens" would show the regime's specific characteristics. In regime 1, the categories "Preface" and "Election Management" have a relative high strengths comparing with other regimes. The relatively high strengths of "Economy" in regime 2 and "Courts" in regime 3 would also reflect the high interest about those issues at that time.

S7 Share of link weights between categories of each regime's CLN

In Figure 2E of the main menuscript, we present the chord diagram which shows the net flow between categories of the total CLN. Likewise, we aggregate all articles into their categories and observe the share of linkweights between categories for different regimes' CLNs. This can be interpreted as the blockmodel representation [8]. We visualize these information onto the

heatmap (Figure F) and found the dominant concentration of linkweights to “Rights and Duties of Citizens” for all regimes.

S8 Prevalence measures of each regime

S8.1 Nodes’ prevalance

A high prevalence of an article in a given regime r means that the article was more predominant than in any other regimes. In the main menuscript, we present this information as the word clouds for better visualizations. In this section, we provide the full information as scatter plots of each regime (Figure G). For all figures, top 3 articles of high prevalence in each regime are presented and these would coincide with the articles with big font sizes in Figure 4 of the main menuscript.

S8.2 Links’ prevalence

As nodes’ prevalence, we can also obtain each link’s prevalence of each regime. In the main menuscript, we show two adjacent regimes (regimes 4 and 5) with markedly different structures within their networks. In this section, we present all the core structure of each regime’s network identified by link prevalence (Figure H).

S9 Time-dependent analysis of the evolution of the network

S9.1 Giant component evolution

The giant component analysis has been a representative approach to study the evolution of the network [9, 10]. Recently, the approach has been found useful to analyze the complex legal system [11]. We observe the giant component size as the event time goes on (Figure I). The event time is defined by one singular legal decision so it corresponds to the link addition to the system.

The giant component emerges at the early stage and grows slowly as time goes on. As we found in the main result, only a few of the preexisting (central) articles were preferentially cited then the cumulative strength distribution becomes fat-tailed. In the middle of the period, the giant component size grows fast again and the period is corresponds to the start of

regime 4. As we described in the manuscript, the high prevalence of "Education" and "Family, mother and health" articles shows the distinctive tendency of the regime, and the characteristics can be also captured in the growth of the giant component size (Figure I).

S9.2 The evolution of the network centrality of categories

Centrality analysis in networks reveals which articles take a central position in the network. First, we observe the evolution of the weighted degrees for each category as time goes on (Figure J).

S9.3 The evolution of the network centrality of articles

For the most microscopic analysis, we present the evolution of the strengths for each article as time goes on (Figure K to P).

References

[1] NATIONAL LAW INFORMATION CENTER, http://www.law.go.kr/eng/engMain.do (Date of access: 14/06/2018).

[2] Constitutional Cours of Korea, http://english.ccourt.go.kr (Date of access: 14/06/2018).

[3] Watts, D. J., and Strogatz, S. H. Collective dynamics of 'small-world' networks. Nature **393**, 440 (1998).

[4] Newman, M. E. J. The structure of scientific collaboration networks. Proc. Natl. Acad. Sci. USA **98**, 404 (2001).

[5] Goh, K.-I., Cusick, M. E., Valle, D., Childs, B., Vidal, M., and Barabási, A.-L. The human disease network, Proc. Natl. Acad. Sci. **104**, 8685 (2007).

[6] Saavedra, S., Reed-Tsochas, F., and Uzzi, B. A simple model of bipartite cooperation for ecological and organizational networks, Nature **457**, 463 (2009).

[7] Serrano, M. A., Boguñá, M. and Vespignani, A. Extracting the multiscale backbone of complex weighted networks. Proc. Natl. Acad. Sci. **106**, 6483 (2009).

[8] Wasserman, S., and Faust, K. *Social network analysis: Methods and applications.* (Cambridge Univ. Press 1994).

[9] Cohen R, Havlin S. Complex Networks: Structure, Robustness and Function: Cambridge University Press; 2010.

[10] Newman MEJ, Strogatz SH, Watts DJ. Random graphs with arbitrary degree distributions and their applications, Physical Review E. 2001; 64(2):026118.

[11] Ruhl JB, Katz DM, Bommarito II MJ. Harnessing legal complexity. Science. 2017; 355(6332): 1377-1378.

f

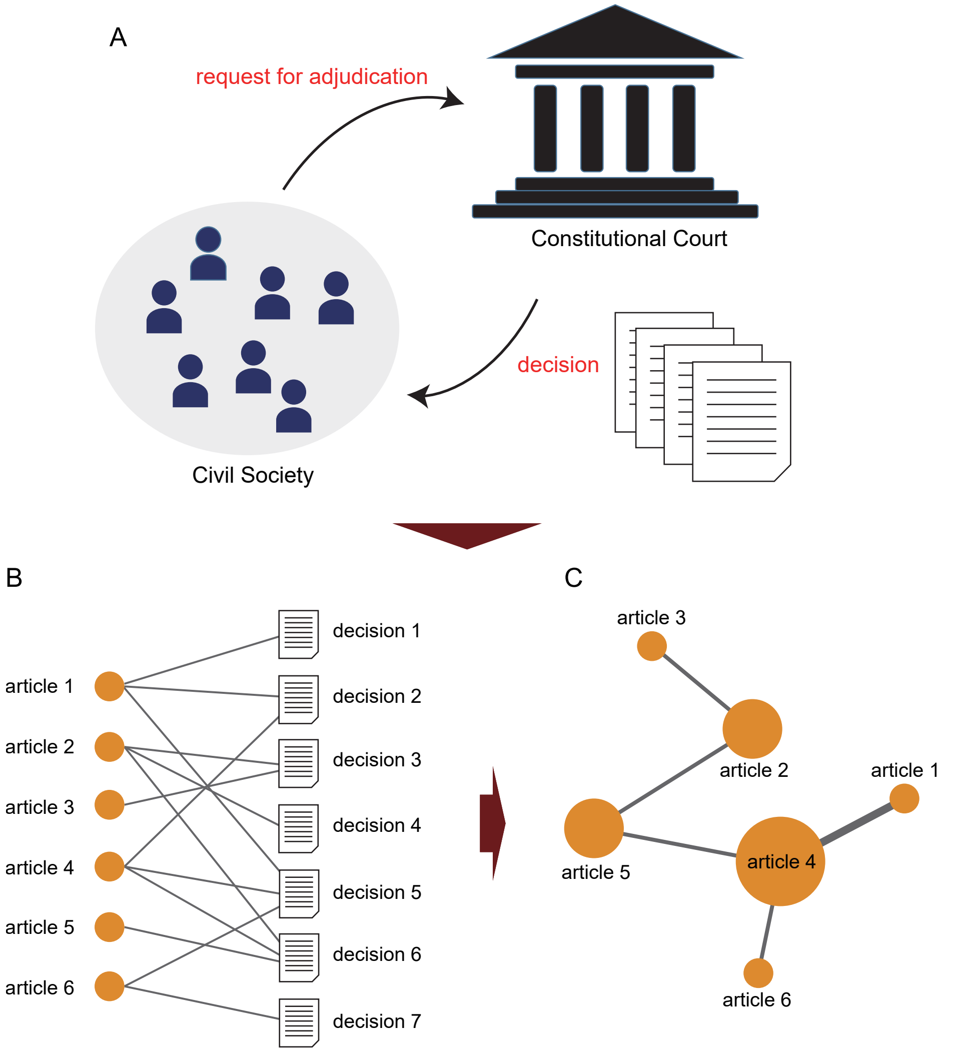

Figure A: (A) The schematic diagram of the constitutional decision process. From all decisions, we construct the bipartite relations among articles and decisions (B) and generate a projection to the articles to obtain the Constitutional Legal Networks (C).

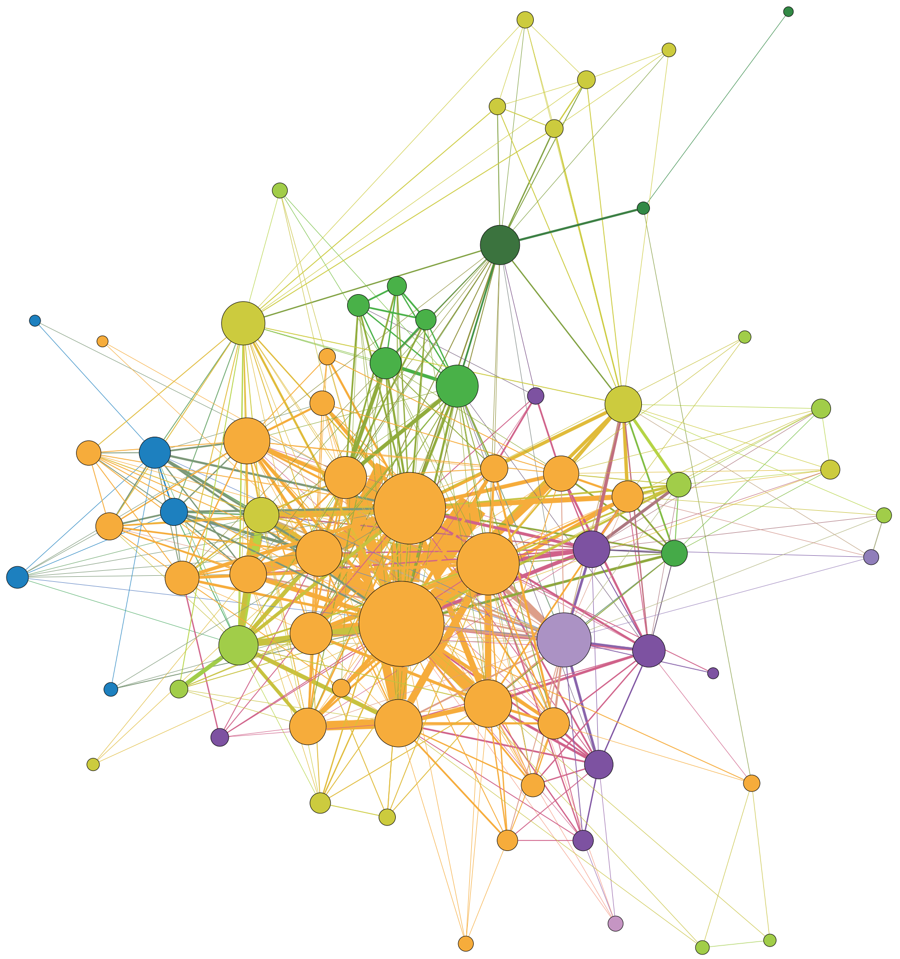

Figure B: The full Constitutional legal network. The size of the nodes is adjusted by their degrees and all the links are presented here. Nodes and links are colored according to the categories in which it is included.

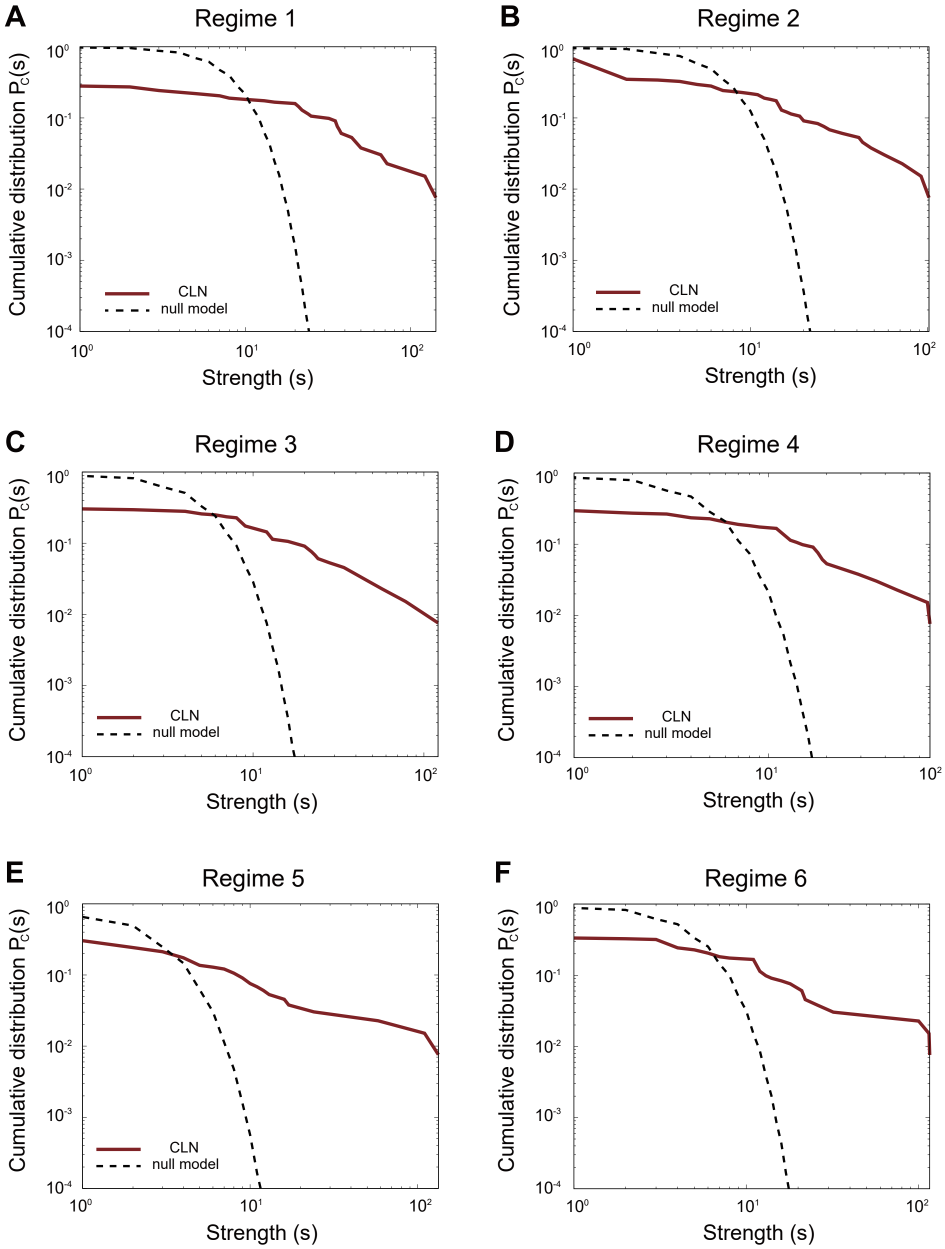

Figure C: The cumulative strength distribution (solid line) of the CLN of regime 1 (A) to 6 (F), respectively. The null model counterparts (dotted line) are presented for the comparison.

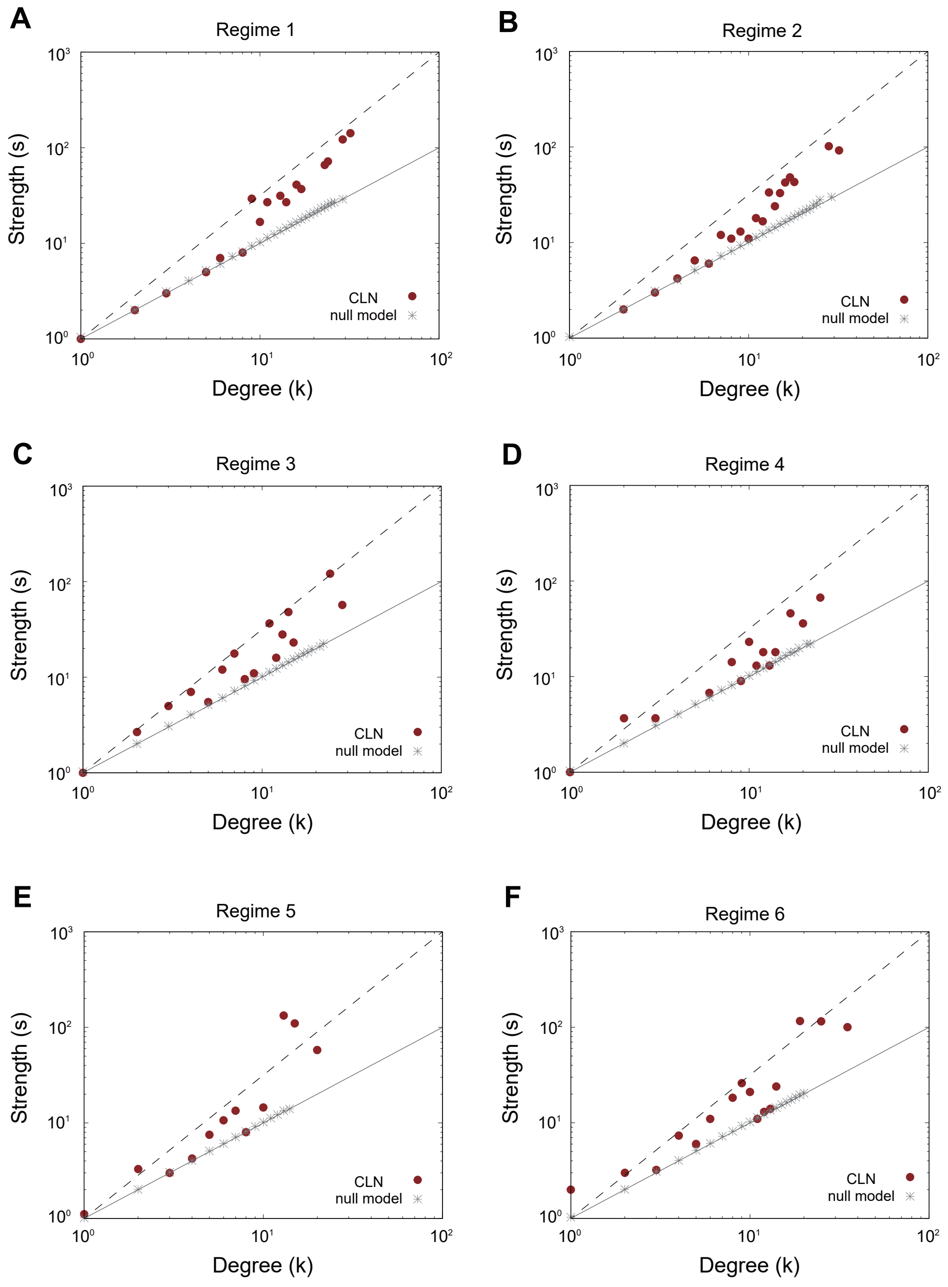

Figure D: The scaling relation between degrees and strengths for each node (red dots) compared with the null model (gray stars) of the CLN of regime 1 (A) to 6 (F), respectively. The dashed (slope of 1.5) and dotted (slope of 1.0) lines are presented as guides. All data is binned for each degree k.

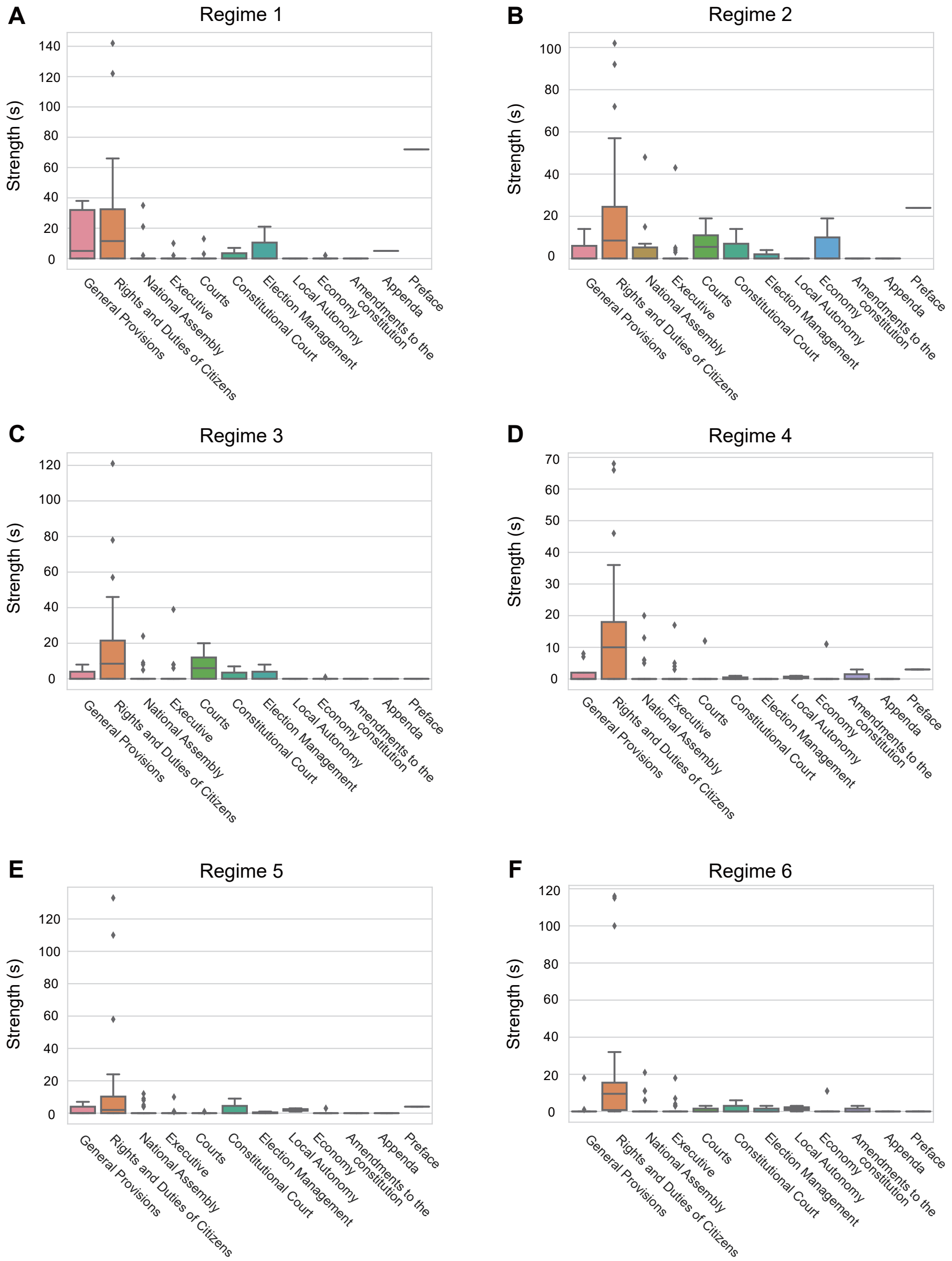

Figure E: Box plots of strengths of articles in each category in regime 1 (A) to 6 (F), respectively.

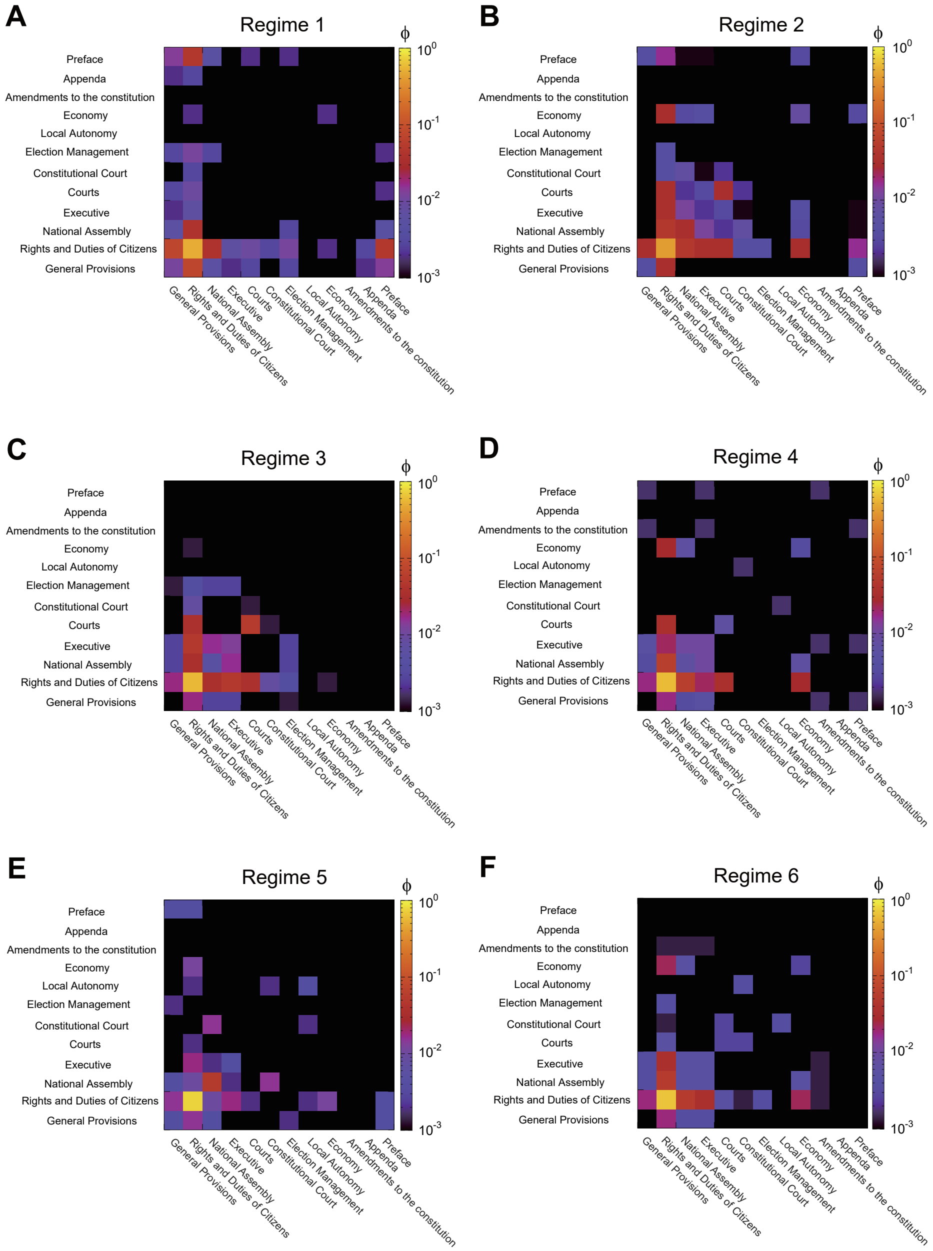

Figure F: Share of link weights between categories for the CLN of regime 1 (A) to 6 (F), respectively. Color bars are logarithmically scaled for better visualization.

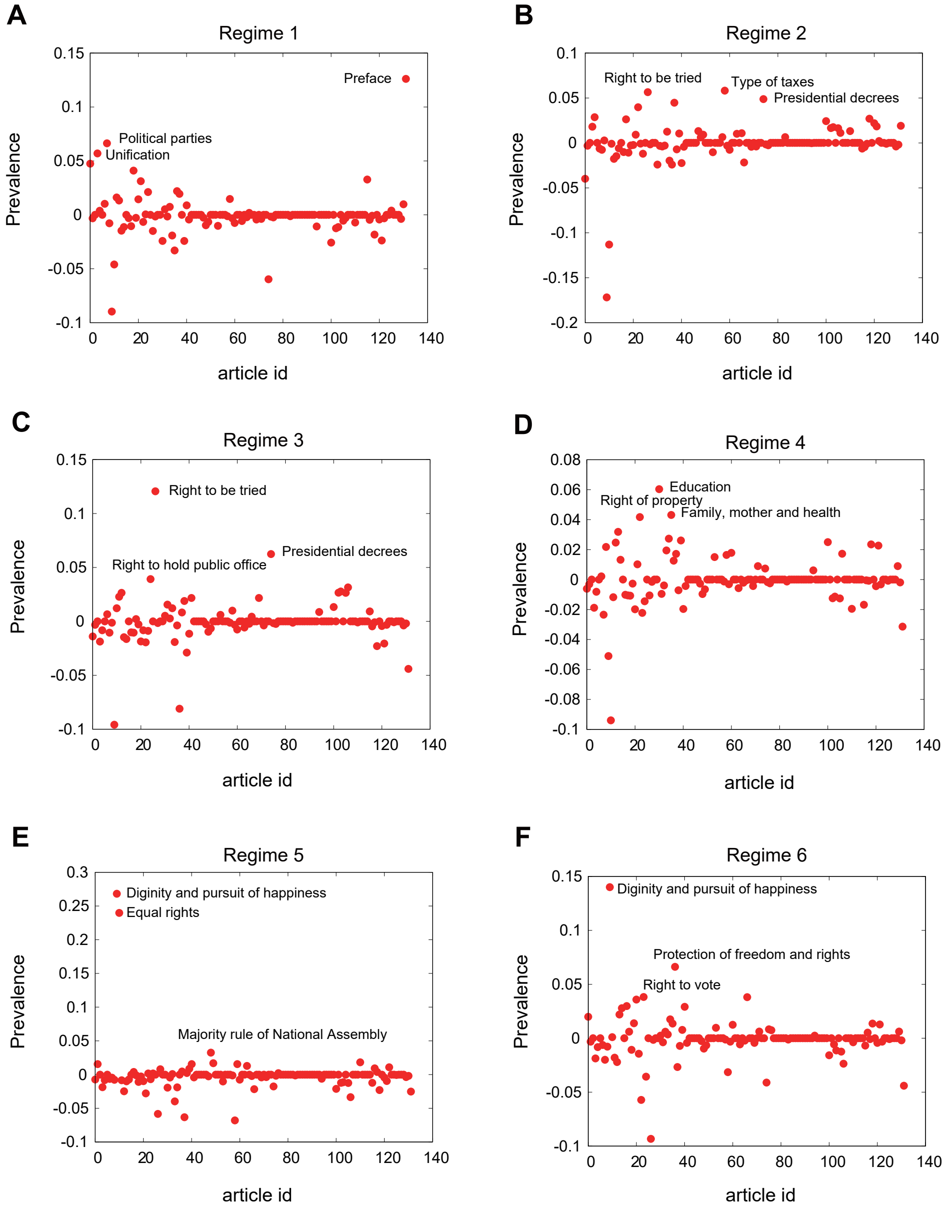

Figure G: Scatter plots of nodes' prevalences for each regime. Top 3 articles of high prevalence in each regime are also presented as texts.

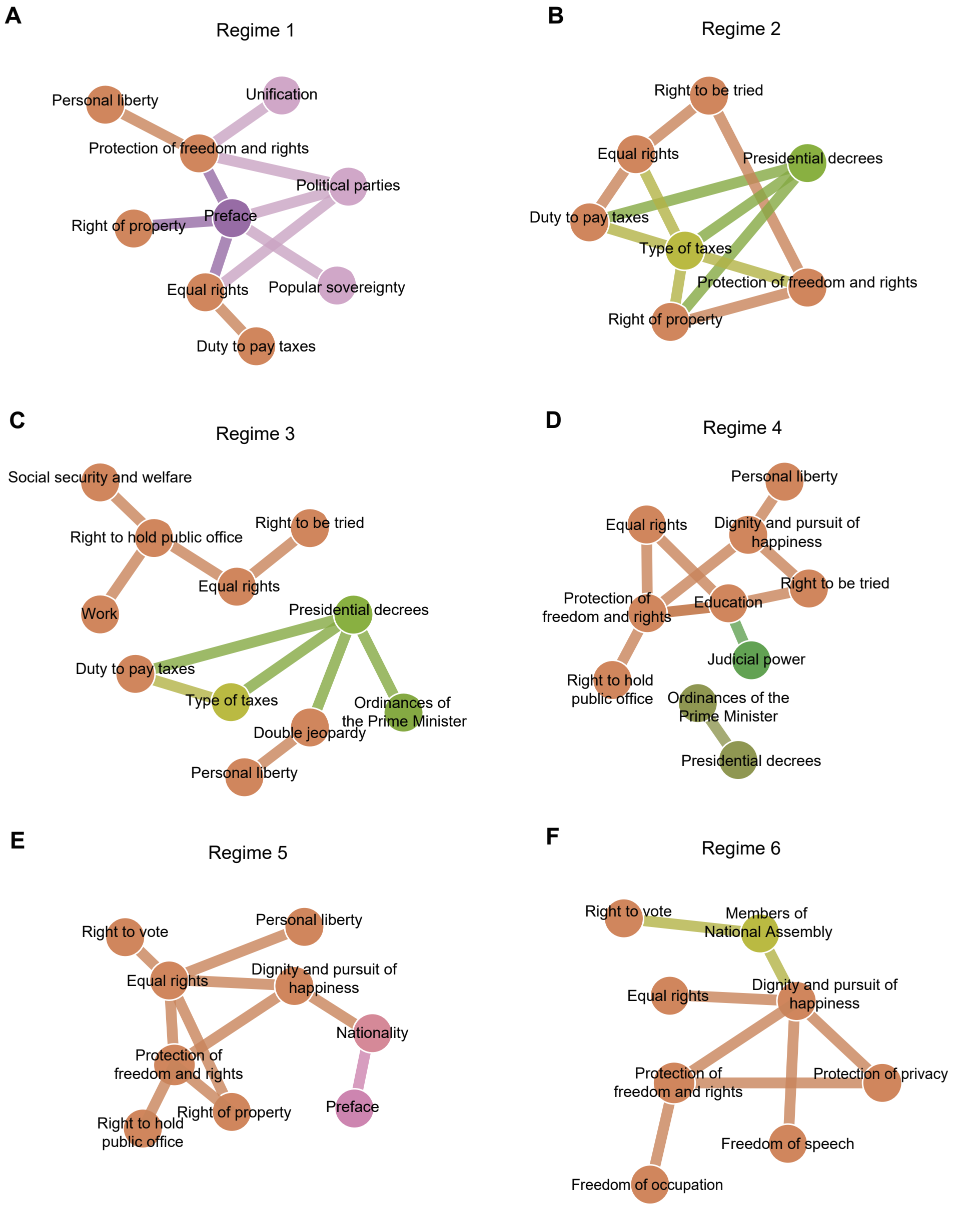

Figure H: The core structure of each regime's network identified by link prevalence. Nodes and links are colored according to the categories in which it is included.

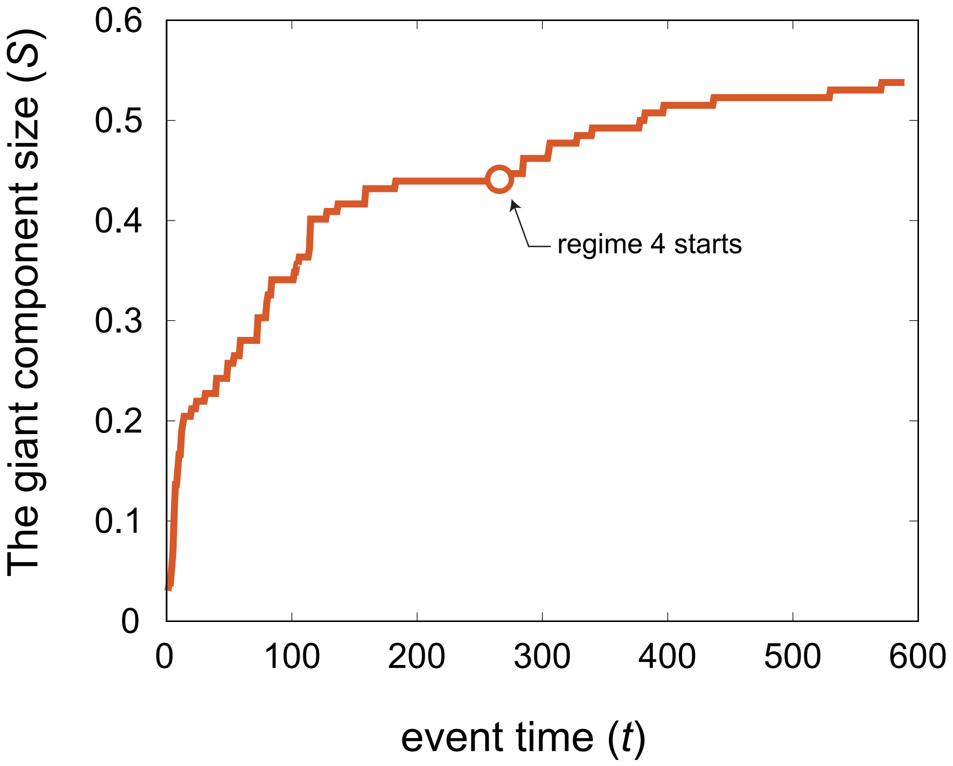

Figure I: Evolution of the giant component as the event time progresses. After the increase of the giant component saturated for a while, it grows fast again when regime 4 starts. In regime 4, several articles like "Education" and "Family, mother and health", which were not paid attention to other regimes, entered the spotlight.

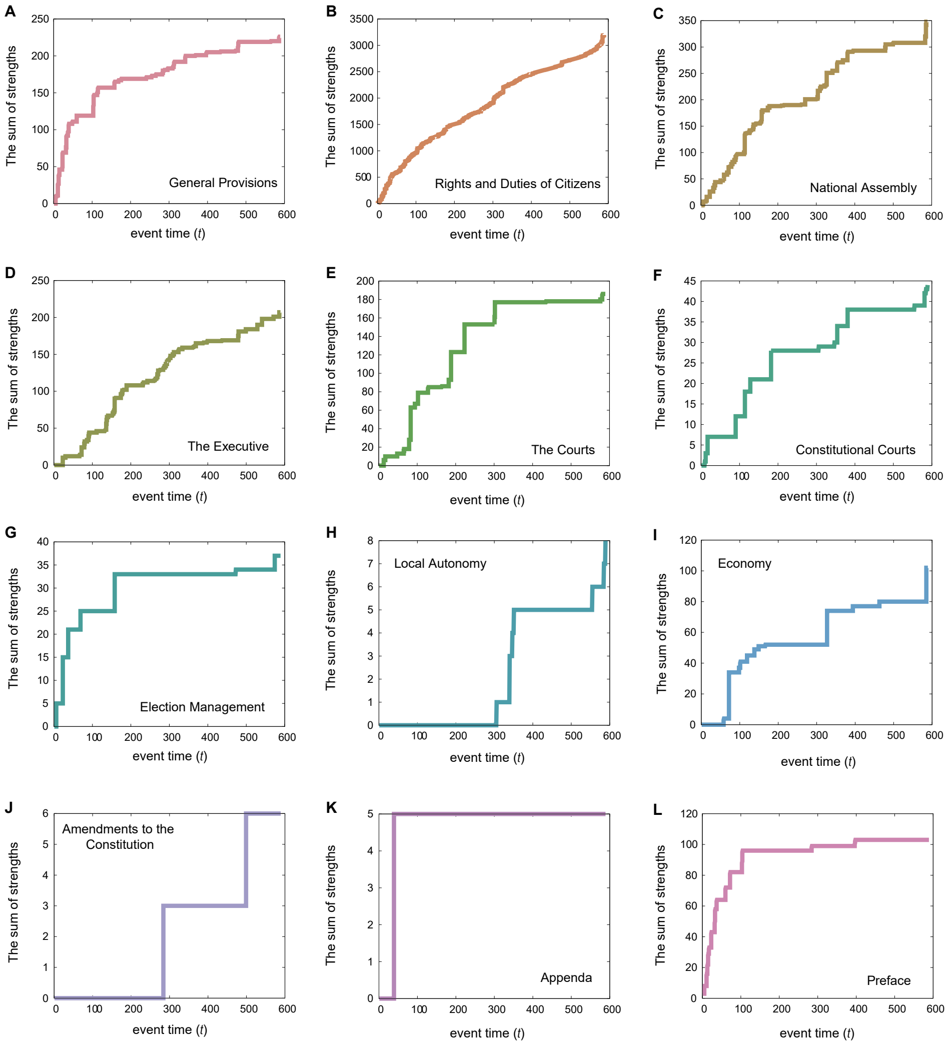

Figure J: Evolution of the sum of strengths of each category.

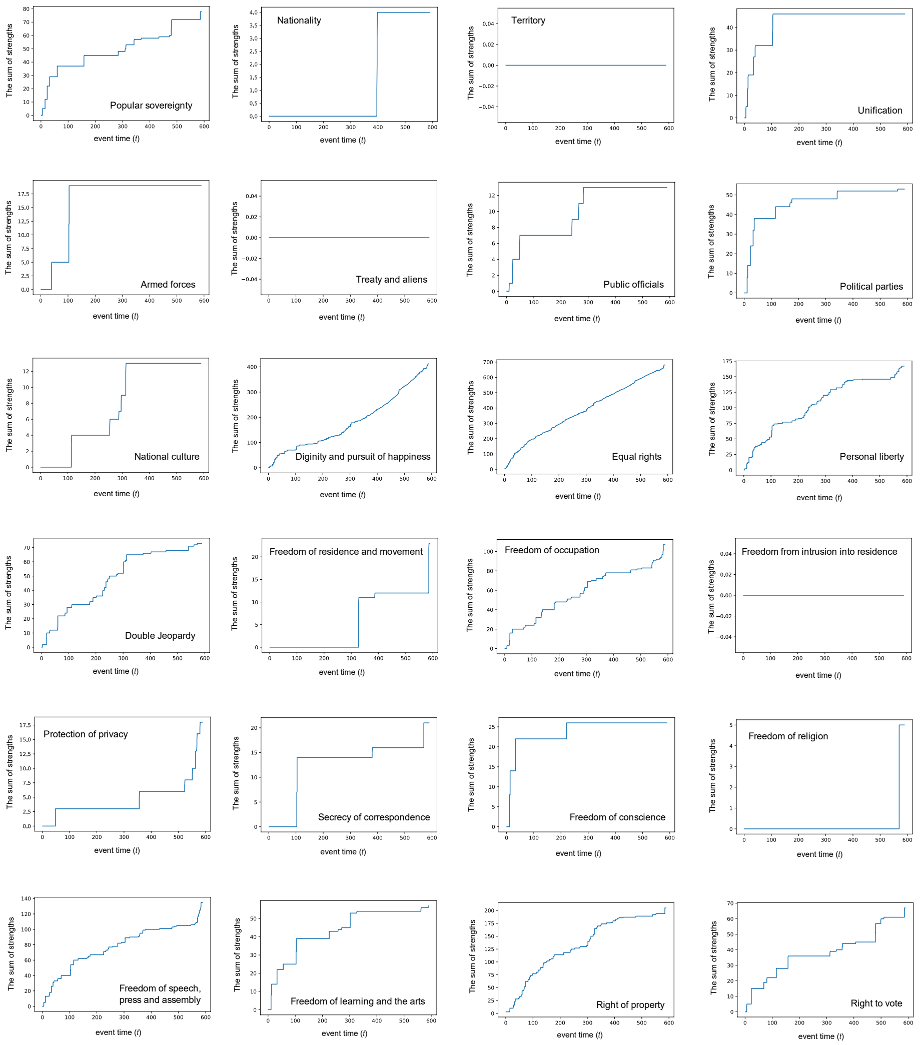

Figure K: Evolution of the sum of strengths of articles 1 to 24.

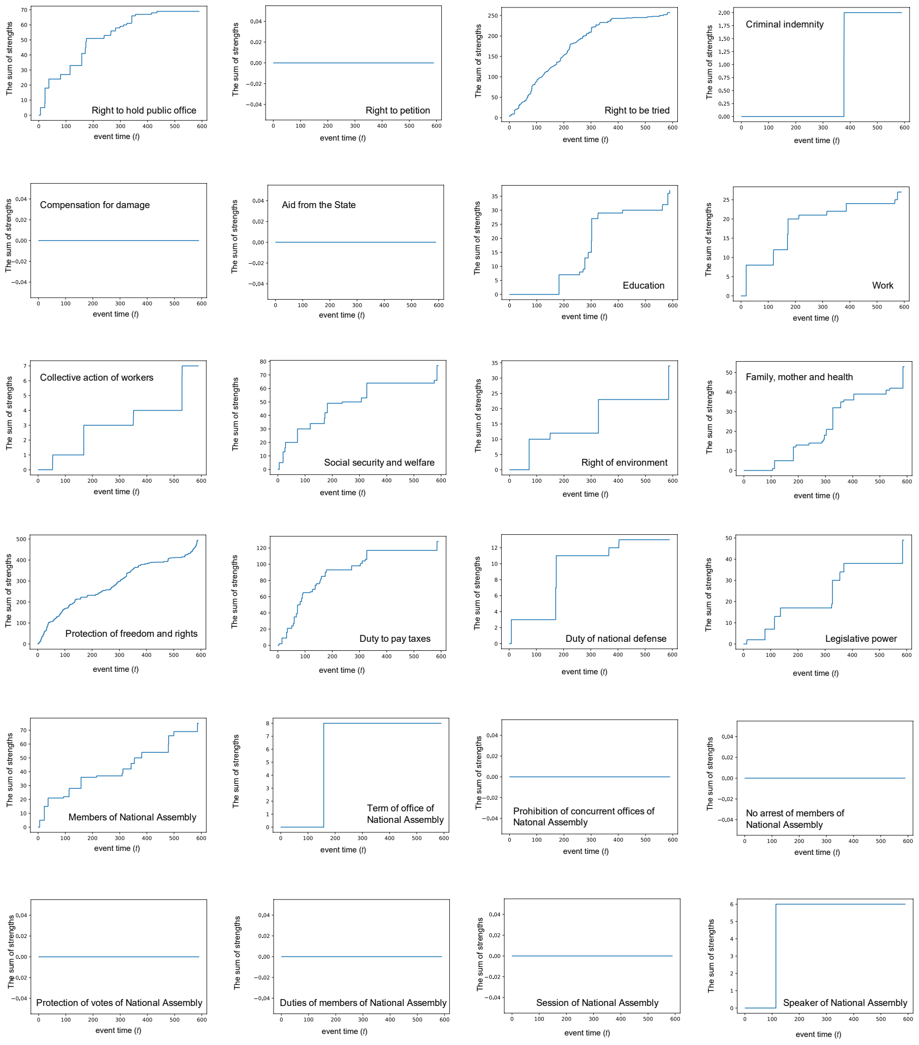

Figure L: Evolution of the sum of strengths of articles 25 to 48.

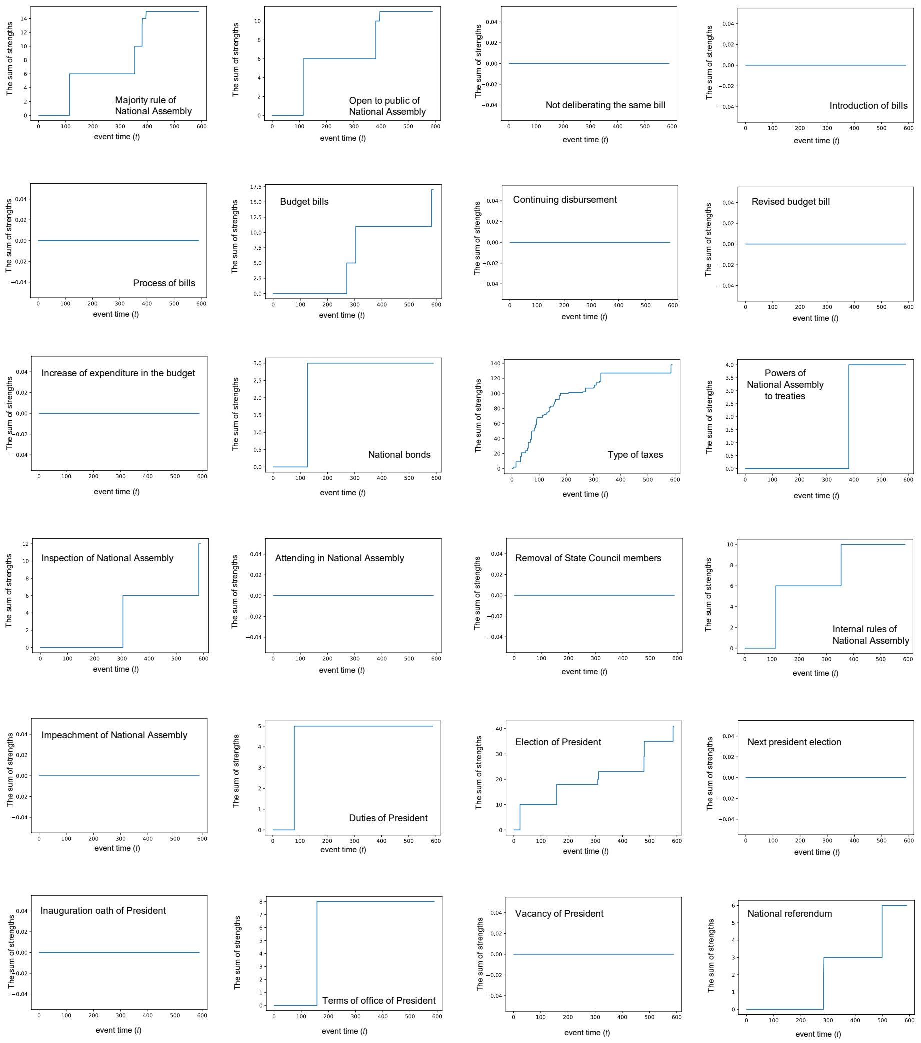

Figure M: Evolution of the sum of strengths of articles 49 to 72.

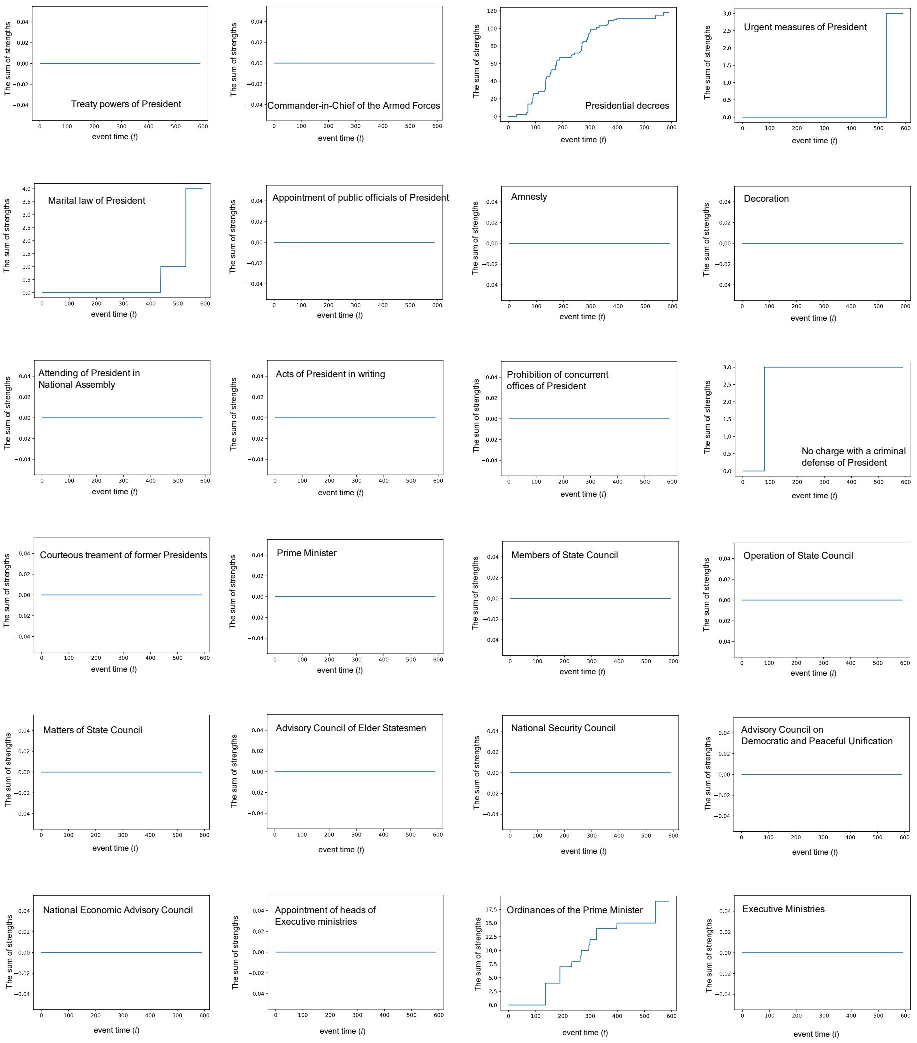

Figure N: Evolution of the sum of strengths of articles 73 to 96.

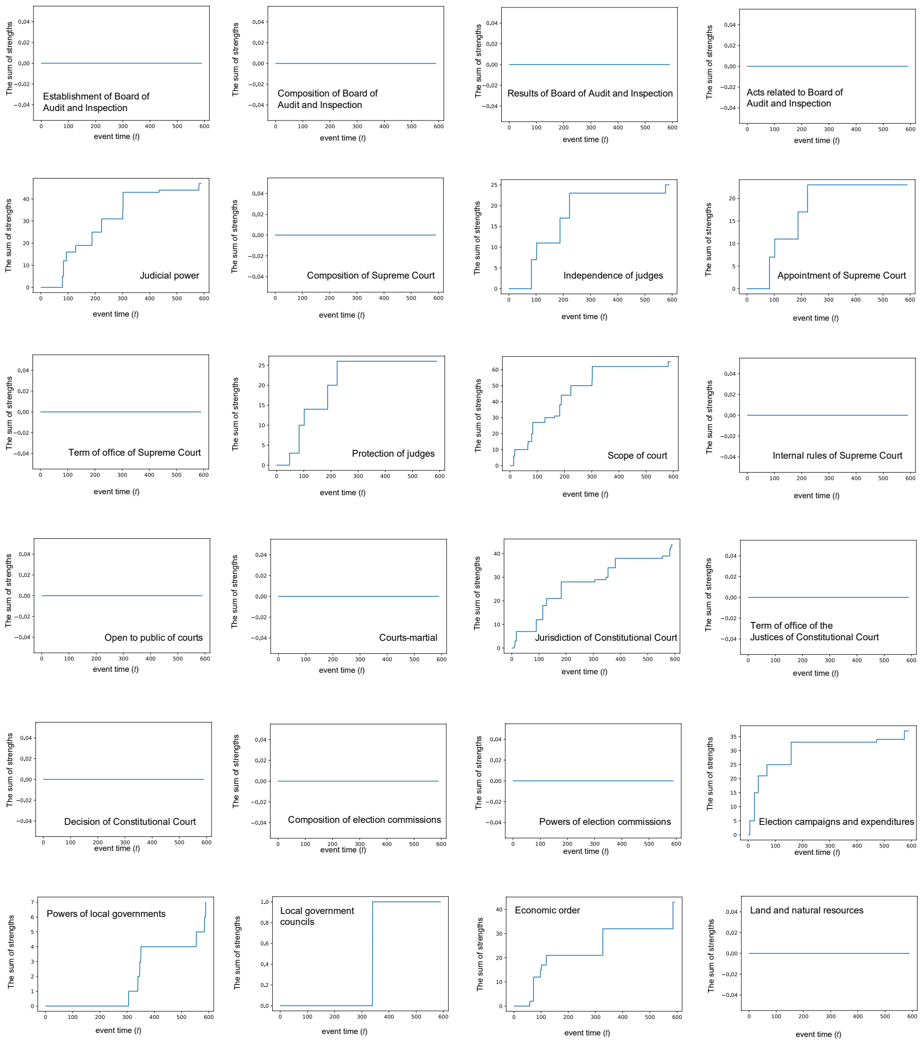

Figure O: Evolution of the sum of strengths of articles 97 to 120.

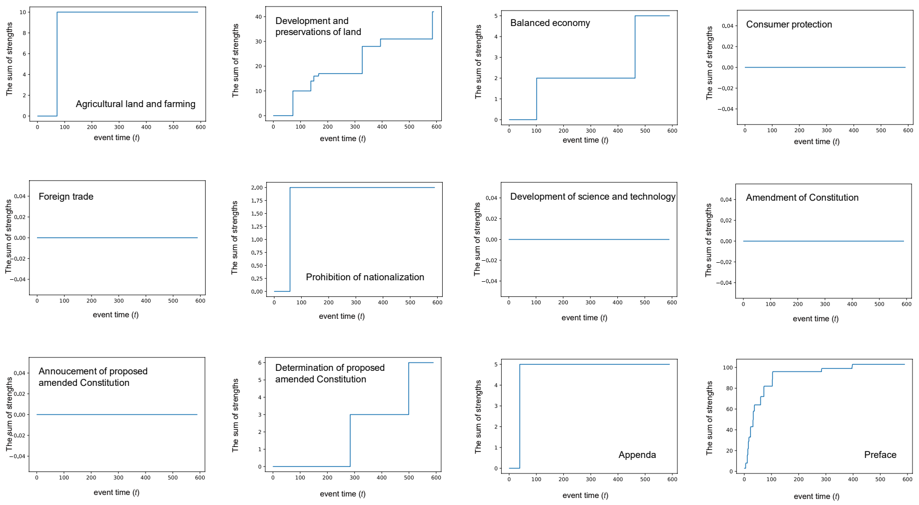

Figure P: Evolution of the sum of strengths of articles 121 to 132.
